# Autism-linked mutations of CTTNBP2 reduce social interaction and impair dendritic spine formation via diverse mechanisms

**DOI:** 10.1186/s40478-020-01053-x

**Published:** 2020-11-09

**Authors:** Pu-Yun Shih, Bing-Yuan Hsieh, Ching-Yen Tsai, Chiu-An Lo, Brian E. Chen, Yi-Ping Hsueh

**Affiliations:** 1 Molecular and Cell Biology, Taiwan International Graduate Program, Institute of Molecular Biology, Academia Sinica and Graduate Institute of Life Sciences, National Defense Medical Center, Taipei, Taiwan, ROC; 2 Institute of Molecular Biology, Academia Sinica, Taipei, Taiwan, ROC; 3 Centre for Research in Neuroscience, McGill University Health Centre, Montreal General Hospital, Montréal, QC H3G 1A4 Canada; 4 Departments of Medicine and Neurology & Neurosurgery, McGill University, Montréal, QC Canada

**Keywords:** Autism spectrum disorder, Cortactin, Dendritic spine formation, F-actin, Microtubule

## Abstract

Abnormal synaptic formation and signaling is one of the key molecular features of autism spectrum disorders (ASD). *Cortactin binding protein 2* (*CTTNBP2*), an ASD-linked gene, is known to regulate the subcellular distribution of synaptic proteins, such as cortactin, thereby controlling dendritic spine formation and maintenance. However, it remains unclear how ASD-linked mutations of CTTNBP2 influence its function. Here, using cultured hippocampal neurons and knockin mouse models, we screen seven ASD-linked mutations in the short form of the *Cttnbp2* gene and identify that M120I, R533* and D570Y mutations impair CTTNBP2 protein–protein interactions via divergent mechanisms to reduce dendritic spine density in neurons. R533* mutation impairs CTTNBP2 interaction with cortactin due to lack of the C-terminal proline-rich domain. Through an N–C terminal interaction, M120I mutation at the N-terminal region of CTTNBP2 also negatively influences cortactin interaction. D570Y mutation increases the association of CTTNBP2 with microtubule, resulting in a dendritic localization of CTTNBP2, consequently reducing the distribution of CTTNBP2 in dendritic spines and impairing the synaptic function of CTTNBP2. Finally, we generated heterozygous M120I knockin mice to mimic the genetic variation of patients and found they exhibit reduced social interaction. Our study elucidates that different ASD-linked mutations of CTTNBP2 result in diverse molecular deficits, but all have the similar consequence of synaptic impairment.

**Electronic supplementary material:**

The online version of this article (10.1186/s40478-020-01053-x) contains supplementary material, which is available to authorized users.

## Introduction

Autism spectrum disorders (ASD) are highly prevalent neurodevelopmental diseases mainly caused by genetic variations [2, 19]. Hundreds of mutated genes have been associated with ASD (https://gene.sfari.org/database/human-gene/). One critical feature of ASD-causative or -linked genes is their roles in controlling synapse formation and synaptic signaling and activity [7, 11, 18], suggesting a crucial aspect of synaptic function in ASD.

Among the various synapse-associated ASD candidate genes, *CTTNBP2* was identified as a strong candidate ASD-linked gene based on several human genetic studies [7, 11, 17, 18]. A study of *Cttnbp2* knockout mice has also indicated that CTTNBP2 regulates social interaction, ultrasonic vocalization and hyperactivity [20], further strengthening the role of CTTNBP2 in ASD. To date, 38 mutations in the *CTTNBP2* gene have been identified in patients with ASD (https://gene.sfari.org/database/human-gene/CTTNBP2#variants-tab). However, how ASD-linked mutations of *CTTNBP2* alter neuronal function to result in autism-like symptoms remains elusive.

Sequence analysis predicted that alternative RNA splicing results in three CTTNBP2 isoforms, i.e. short, long and intron forms, but the protein products of the long and intron forms of CTTNBP2 are not detectable in the brain [5, 14]. Instead, a protein species of ~ 90 kDa, i.e. the short form, is the only detectable protein product of *CTTNBP2* in the brain [5, 14, 20, 21]. Therefore, mutations within the CTTNBP2 short form, hereafter simply denoted CTTNBP2, are expected to be more relevant to ASD, so we have focused on seven such ASD-linked mutations in this report.

**Fig. 1:**
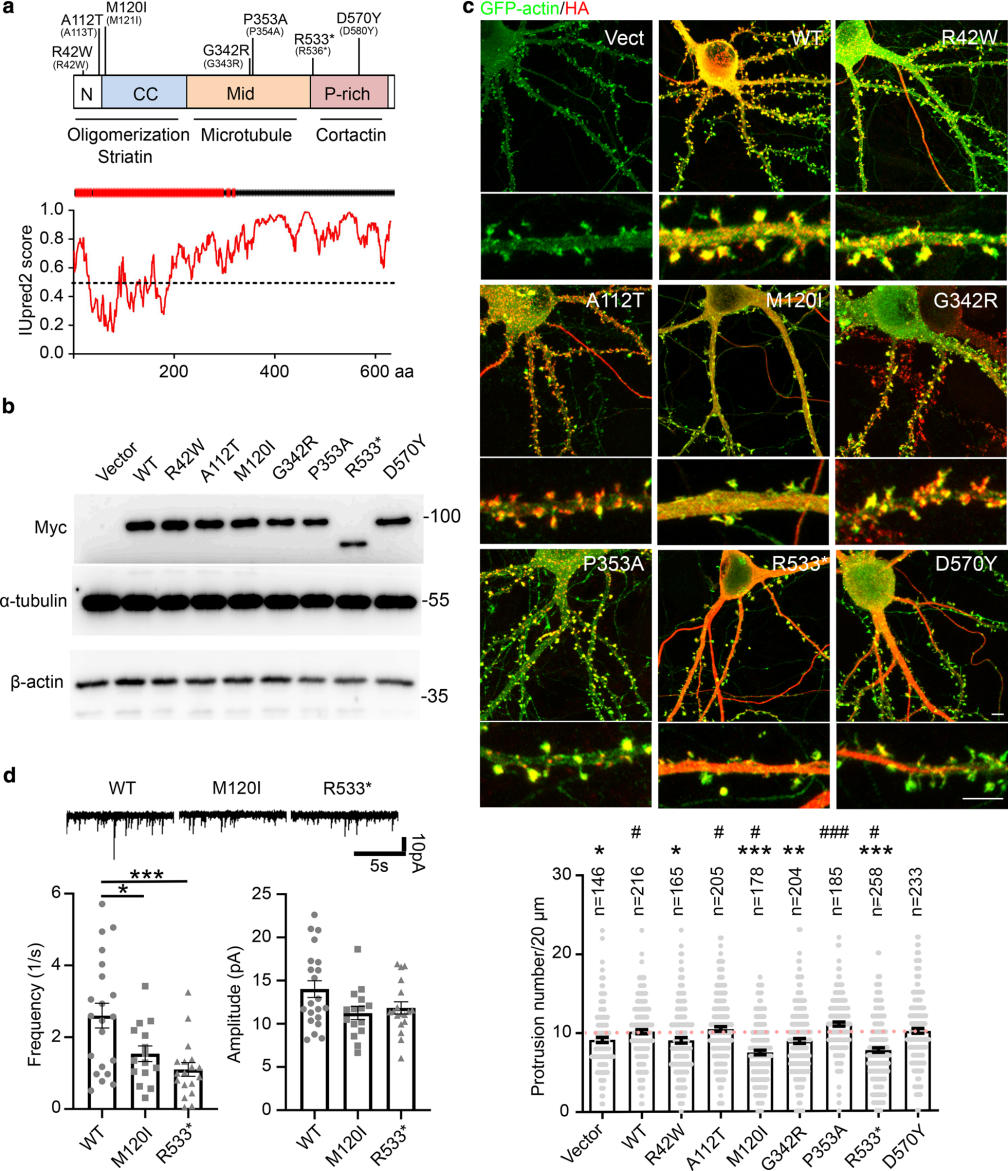
M120I and R533* mutants reduce dendritic spine density and mEPSC frequency. **a** Upper: Schematic of the protein structure of CTTNBP2. Seven human ASD-linked mutations (in parentheses) and their corresponding residues in mouse protein are indicated. Binding partners of each domain are noted below. N, N-terminal region; CC, coiled-coil domain; Mid, middle region; P-rich, proline-rich domain. Middle: Predicted secondary structure of CTTNBP2 by I-TASSER (https://zhanglab.ccmb.med.umich.edu/I-TASSER). Red part represents alpha-helix, and black represents coil. Lower: IUpred2A (https://iupred2a.elte.hu) prediction of disordered regions of CTTNBP2. Higher score indicates a higher degree of disorder. **b** Confirmation of expression of Myc-tagged CTTNBP2 ASD-linked mutant proteins in COS1 cells. β-actin and α-tubulin were used as internal controls. **c** The effect of CTTNBP2 ASD-linked mutations on dendritic spine density. M120I and R533* mutation showed the most dramatic reduction in dendritic spine density. Representative image of cultured hippocampal neurons that express HA-tagged WT or ASD-linked mutant proteins (viewed in red). Dendritic morphology was outlined by GFP-actin (visualized in green). Quantification results of dendritic spine density are shown. The quantification results of vector, WT and R533* have been reported previously [20]. Since those three groups of experiments were performed at the same time as the experiments on the other mutant proteins targeted for this study, we compare all mutants together here. **d** M120I and R533* mutants reduce mEPSC frequency but not amplitude. Data represent mean ± SEM. Each dot in (**c**) and (**d**) indicates the individual result for a dendritic segment and neuron, respectively. The results were collected from three (**c**) and ten (**d**) independent cultures. **c** Kruskal–Wallis test with Dunn’s multiple comparison test were used to compare mutant protein with the wild-type (WT) group (*) or vector control (#). **d** One-way ANOVA was used for analysis. See Additional file 2: Table S1 for all statistical data and exact sample size for each group. * or #*P* < 0.05; **P < 0.01; *** or ###*P* < 0.001. Scale bar, 5 μm

CTTNBP2 consists of an N-terminal coiled-coil domain for homo- and hetero-oligomerization, a middle region for microtubule binding, and a C-terminal proline-rich domain that interacts with cortactin (Fig. 1a) [4, 14, 21]. CTTNBP2 modulates neuronal morphogenesis by regulating actin and microtubule dynamics [9]. In immature neurons, CTTNBP2 associates with microtubule and promotes microtubule stability along dendrites. This stabilization contributes to dendritic arborization [21]. As neurons mature, CTTNBP2 shifts from the dendritic shaft into dendritic spines where it facilitates synaptic targeting of cortactin [5]. This role in synaptic targeting is critical for dendritic spine formation and maintenance because both *Cttnbp2* knockdown and expression of a CTTNBP2 mutant that cannot interact with cortactin results in reduced dendritic spine density and size [5]. Apart from its contribution to cytoskeleton dynamics, proteomic analysis has further indicated that CTTNBP2 controls the synaptic expression of more than a hundred proteins, including SHANK2, SHANK3, striatin (STRN), and RAC3 [20]. These synaptic proteins may also contribute to the function of CTTNBP2 in neurons.

In this study, we utilized cultured neurons and knockin mice carrying ASD-linked mutations in CTTNBP2 to investigate how these mutations alter CTTNBP2 activity and influence neuronal morphology and function. We found that although different mutations impact divergent molecular functions of CTTNBP2, they all modulate dendritic spine density. Our study sheds light on the multiple regulatory roles of CTTNBP2 in dendritic spine formation and provides mechanistic evidence for how *CTTNBP2* mutations contribute to ASD etiology.

## Materials and methods

### Experimental design

Since seven ASD-linked mutations in human CTTNBP2 are conserved among human, rat and mouse (Additional file 1: Fig. S1), we used rat cultured hippocampal neurons and knockin mice to analyze how ASD-linked mutations influence the function of CTTNBP2 in terms of dendritic spine density, neuronal activity and mouse behaviors. We primarily overexpressed ASD-linked mutations in wild-type (WT) hippocampal neurons and used heterozygous knockin mice to mimic monoallelic mutations of *CTTNBP2* in patients. Based on our analysis of dendritic spine formation in cultured neurons, we then focused on three mutations, i.e. M120I, R533* and D570Y, because they altered the subcellular distribution of CTTNBP2 and impaired dendritic spine formation. A series of biochemical and cell biology approaches were employed to dissect the molecular defects caused by ASD mutations. Neuronal morphology and mouse behaviors of ASD mutant mice were then investigated to elucidate the physiological impact of ASD-linked mutations in the *Cttnbp2* gene in vivo. All morphometric, electrophysiological and behavioral analyses were conducted blind by relabeling the samples before analysis by another member in the laboratory. All statistical methods and results are summarized in Additional file 2: Table S1, Additional file 3: Table S2.

### Animals

All the animal experiments in this study were performed with the approval of the Academia Sinica Institutional Animal Care and Utilization Committee (Protocol # 12-10-414 and 11-12-294). Animal housing and handling were conducted according to the guidelines of the Council of Agriculture Guidebook for the Care and Use of Laboratory Animals. For primary hippocampal neuron culture, pregnant rats were sacrificed by CO_2_ inhalation. Embryonic day E18.5 fetal pups of both sexes were isolated and sacrificed by decapitation. Male mice at the age of 2-3 months were used for behavioral assays. All animals were housed and bred in the animal facility of the Institute of Molecular Biology, Academia Sinica, under controlled humidity and temperature and a 12 h light/dark cycle (light off at 20:00). Animals accessed water and food (#5K54, LabDiet) ad libitum. All genetically modified mice had been backcrossed to WT C57BL/6 mice for more than six generations to minimize off-target effects of CRISPR/Cas9 editing.

#### CRISPR/Cas9 technology for introducing ASD-associated mutations into Cttnbp2

A paired-nicking approach [22, 23] was applied to insert mutation sequences into exon 4 of *Cttnbp2*. For the *Cttnbp2* M120I mutation, the paired guide RNAs (5′- AGAAAGGATGTCCGCACAGC and 5′- AGTGGGCCATGACAGCTTCA) and a single-stranded DNA template (ssODN: 5′- TGTGAGCCAGTTCTGCTGTTCCTTGCTATTATGGGAAAAACAGATGACGTCTCAGTACCTTTTTTTGTCTGCTCTCAGCGGCCACCAGCTGTGCGGACATCCTTTCTTGtATTTTTCTaCAGTGGGCCATGACAGCTTCAAGGATGGAGAGTGGATTGGTGCAGACTGGCTTCTCTTTGTCACCAGGACCTGCTTCATAG) were used. Two nucleotides (in lower case) were changed in order to alter the coding sequences of methionine to isoleucine at residue 120 (shown in bold and underlined) and to disrupt the PstI site (underlined) without changing the encoded amino acid.

For the *Cttnbp2* D570Y mutation, a guide RNA (5′-ATCAACTTTGGCCCCTGCAT) and a single-stranded DNA template (ssODN: 5′- GGAAATCCTCCTCCTATCCCTCCCAAAAAGCCAGGGCTCTCCCAAACTCCTTCTCCGCCACACCCCCAACTGAGGGCCTCCAATGCAGGGGCCAAAGTTtATAACAAgATTGTGGCTTCACCTCCCTCTACTTTGCCACAAGG) were used. Two nucleotides (lower case) were changed in order to alter the coding sequence from aspartic acid to tyrosine at residue 570, and to create a PsiI site and disrupt the MluCI site without changing the encoded amino acid, respectively.

A T7 promoter sequence (5′- TTAATACGACTCACTATA) was added upstream of gRNA sequences and a partial tracrRNA sequence (5′- GTTTTAGAGCTAGAAATAGC) was added downstream of the gRNA sequence. The oligo was annealed with reverse tracrRNA (5′-TTTAAAAGCACCGACTCGGTGCCACTTTTTCAAGTTGATAACGGACTAGCCTTATTTTAACTTGCTATTTCTAGCTCTAAAAC) and PCR-amplified using Phusion DNA Polymerase (Thermo-Fisher Scientific) according to the manufacturer’s instructions. The amplified product was purified using QIAquick PCR Purification Kit (28106, Qiagen) and it served as the in vitro transcription template.

The sgRNAs were synthesized using HiScribe™ T7 Quick High Yield RNA Synthesis Kit from NEB (2050S, NEB). Cas9A^D10A^ mRNA was synthesized using mMESSAGE mMACHINE T7 Ultra Kit (AM1345, Thermo-Fisher Scientific) using pCAG-T3-hCasD10A-pA (#51638, Addgene) as template, purified using MEGAclear Transcription Clean-up Kit (AM1908, Thermo-Fisher Scientific), and eluted with injection buffer (10 mM Tris–HCl pH7.2 and 0.1 mM ethylenediaminetetraacetic acid (EDTA)). The quality and quantity of RNAs were analyzed using a NanoDrop ND-1000 (Thermo-Fisher Scientific). To extract genomic DNA, ~ 3 mm of mouse tail was cut and lysed in 600 μl 50 mM NaOH and boiled for 30 min. After complete tissue lysis, samples were cooled down briefly on ice before adding 60 μl of 1 M Tris–Cl pH 8.0 to neutralize the pH. Around 0.5-1 μl of DNA was used for PCR reaction. Primer sequences to amplify the M120I mutant allele by PCR were 5′-ATGGCTTTCCAGGCTTGTCAG-3′ and 5′-AGCCCACTCCCACCAAAACTA-3′. To amplify the D570Y mutant allele by PCR, we designed the primer set: 5′-GCCAAGCAGCTAGCTCGGAATAC-3′ and 5′-GTTCAGTCCAGGGGTTCCAGCAG-3′. To distinguish M120I and D570Y alleles, we digested the PCR product by *PstI* (NEB) and *MluCI* (NEB), respectively.

### Plasmids

For immunostaining and biochemical analysis, the GW1-HA-CTTNBP2, GW1-myc-CTTNBP2, GW1-myc-NCC, and GW1-myc-Mid constructs were described previously [4, 5, 21]. For mEPSC recording (Fig. 1d), pCAG-GFP-P2A was constructed by inserting *SuperfolderGFP* and *P2A* sequences into an empty pCAG vector [13]. The original superfolder GFP was obtained from [15], the P2A sequence is from [13], and the empty pCAG vector was ordered from Addgene. HA-tagged *Cttnbp2* was PCR-amplified using the primer set 5′- GCGATATCTTAGGGAGGGTG-3′ and 5′- CCAGATATCATGTACCCATATGAC -3′ and cloned into pCAG-GFP-2A vector plasmid at the EcoRV site. Myc-cortactin was a generous gift from Dr. Morgan Sheng at MIT. To construct the GFP-P-rich domain, the P-rich domain was PCR-amplified using the primer set 5′-GGAATTCCGAACCGGTTTAAAG-3′ and 5′-GGAATTCTTAGGGAGGGTG-3′ and cloned into pEGFP-C2 plasmid at the EcoRI site. Site-directed mutagenesis using PCR was performed to construct ASD-associated *Cttnbp2* mutant constructs. Sequences of all primer pairs (from 5′ to 3′) are as follows (uppercase letters indicate mutated residues):

**Table:** 

BP2 R42W-F	cgctcagcaaatcagagctgTggatgctccttagcgtgatg
BP2 R42W-R	catcacgctaaggagcatccAcagctctgatttgctgagcg
BP2 A112T-F	ccactctccatccttgaaActgtcatggcccactgag
BP2 A112T-R	ctcagtgggccatgacagTttcaaggatggagagtgg
BP2 M120I-F	catggcccactgcagaaaaatAcaagaaaggatgtccgcac
BP2 M120I-R	gtgcggacatcctttcttgTatttttctgcagtgggccatg
BP2 G342R-F	tagttcccacaaacacaaaaAggaatgtgggccccagtgcc
BP2 G342R-R	ggcactggggcccacattccTttttgtgtttgtgggaacta
BP2 P353A-F	ccagtgccctgctgattagaGcaggtattgataggcagtct
BP2 P353A-R	agactgcctatcaatacctgCtctaatcagcagggcactgg
BP2 R533X-F	taaagactcccggggcagcaTgagttgacagaggaaatcctcc
BP2 R533X-R	ggaggatttcctctgtcaactcAtgctgccccgggagtcttta
BP2 D570Y-F	ccaatgcaggggccaaagttTataacaaaattgtggcttc
BP2 D570Y-R	gaagccacaattttgttatAaactttggcccctgcattgg

### Antibodies

The following antibodies and working concentration were used in this study: anti-CTTNBP2 (A5, A7, and 9W, rabbit, homemade, 0.5 μg/ml) [5, 20], anti-Myc tag (9B11, Cell Signaling Technology, 1/1000 for staining; 06-549, Millipore, 1 μg/ml), anti-HA tag (3F10, Roche, 0.5 μg/ml), anti-HA tag (Y-11, Santa Cruz Biotechnology, 0.5 μg/ml), anti-GFP (ab13970, Abcam, 0.5 μg/ml), anti-αtubulin (B-5-1-2, Sigma-Aldrich, 1 μg/ml), anti-acetyl tubulin (6-11B-1, Sigma-Aldrich, 1 μg/ml), anti-βactin (AC-74, Sigma-Aldrich, 1/1000), anti-cortactin (H-191, Santa Cruz Biotechnology, 0.5 μg/ml), anti-FOS (#2250, clone 9F6, Cell Signaling Technology, 1/200), anti-mouse HRP (NA931, GE Healthcare, 1/5000), anti-rabbit HRP (NA934, GE Healthcare, 1/5000), anti-chicken Alexa Fluor 488 (A-11039, Invitrogen, 1 μg/ml), anti-mouse Alexa Fluor 555 (A-21424, Invitrogen, 1 μg/ml), anti-rat Alexa Fluor 594 (A-21209, Invitrogen, 1 μg/ml), and anti-rabbit Alexa Fluor 647 (A-21244, Invitrogen, 2 μg/ml).

### Preparation and transfection of cultured primary hippocampal neurons

Preparation of primary rat hippocampal culture was described previously [5, 21]. Briefly, hippocampi were carefully collected at embryonic day E18.5 and digested with papain solution [0.6 mg/ml papain, 0.5 mM EDTA, 1.5 mM CaCl_2_, 0.06% DNase I, 0.2 mg/ml cysteine] at 37 °C for 25 min. The papain solution was removed and the digested hippocampi were gently washed with Hank’s balanced salt solution (HBSS) buffer. To dissociate the cells, the digested hippocampi were gently pipetted. The cell suspension without debris was then transferred to a new tube and centrifuged at 900 rpm for 5 min to collect dissociated cells. The cell pellets were re-suspended and cell density was determined. For a 12-well plate, 2 × 10^5^ cells/well were seeded on a polylysine-coated glass coverslip.

### Cresyl violet staining

Fifty-μm-thick brain sections were mounted onto glass slides coated with 0.5% gelatin and air-dried. The sections were then stained with cresyl violet solution (0.1% cresyl violet in 1% acetic acid) and destained several times with 70% ethanol until the signal was clear. The sections were serially dehydrated with 70%, 90% and 100% ethanol and then xylene for mounting using Permount mounting medium (Fisher Scientific).

### Immunostaining

For neuron morphology analysis, rat primary cultured hippocampal neurons were fixed by 4% paraformaldehyde with 4% sucrose in phosphate-buffered saline (PBS) for 10 min and permeabilized by 0.2% Triton X-100 in PBS for 10 min. After washing with PBS, the sections were blocked with 10% Bovine serum albumin (BSA) for 30 min in room temperature, and incubated with primary antibodies in 3% BSA overnight in 4 °C. After washing with PBS, the coverslips were incubated with secondary antibodies 3% BSA for 1 h in room temperature. For C-FOS staining, two hours after the reciprocal social stimulation test, the brain was fixed with 4% paraformaldehyde in PBS. Fifty μm-thick brain sections were treated with 1% H_2_O_2_ in Tris–Cl buffer, pH 7.6, for 30 min and permeabilized with 0.05% Tween-20 in PBS for 15 min. After washing with PBS, the sections were blocked with TNB buffer (0.5% blocking reagent in PBS, TSA Fluorescein System Kit, No.1715186, Perkin Elmer) for 1 h and then incubated overnight with primary antibody in TNB buffer at 4 °C. After washing with 0.05% Tween-20 in PBS, sections were incubated with biotinylated goat anti-rabbit IgG secondary antibody (1/200, vectastain, Vector Laboratories) in TNB buffer for 2 h. The immunoreactivity was developed using Vectastain Elite ABC Kit (Vector Laboratories) based on the manufacturer’s instructions. For immunofluorescence staining, brain sections were incubated with primary antibody as described above, followed by incubation with the secondary antibodies conjugated with Alexa Fluor-488, -555, -594, and/or -647 (Invitrogen) for 2 h.

### Microscopy and morphometry analyses

True-color imaging (for C-FOS staining and Cresyl violet stain) was performed using an upright microscope (Microscope Axio Imager M2, Carl Zeiss) equipped with a 10 ×/NA 1.4 oil (Plan-Apochromat, Carl Zeiss) objective lens, and AxioCam (Carl Zeiss) and Zen 2011 software (Carl Zeiss). Shading correction and white balance was applied to correct the signal. For cresyl violet staining, the images were tiled up to acquire entire sections. Fluorescence images were captured using a confocal microscope (LSM700, Carl Zeiss) equipped with a 63 ×/NA 1.4 oil objective lens (Plan-Apochromat, Carl Zeiss) and Zen 2009 (Carl Zeiss) at room temperature. To analyze spine morphology of hippocampal CA1 in vivo, *Cttnbp2* mutant mice were crossed with Thy1-YFP transgenic mice (#003782, Thy1-YFP-H, The Jackson Laboratory) [8]. The first branch of the apical dendrite of CA1 pyramidal neurons was selected for analysis. A 15-μm-long dendritic fragment 5 μm distant from the branch point was used to determine the density and length of spines and the width of spine heads. The Z-series images were captured at 0.2 μm intervals with the “Region” function in Zen 2009 (Carl Zeiss) and processed using the “maximum projection” function. Quantifications were performed using ImageJ. The spine density of two or three dendritic segments from the same neuron was averaged to represent the density of each neuron. Ten neurons were imaged from each animal and at least three mice were used for each group of an experiment.

For cultured hippocampal neurons, we focused on a 20-μm-long segment of the primary dendrite starting 20 μm away from the soma to measure dendritic spine density and the synaptic distribution of CTTNBP2 and cortactin. At least two clearly recognized dendrites were quantified and averaged (using blinded sample relabeling) for each neuron to represent the spine density or protein distribution of each neuron. The sample sizes of examined neurons and dendritic segments are summarized in Additional file 2: Table S1. To quantify the synaptic enrichment of cortactin, we quantified the cortactin signal in individual spines and normalized them with the average signal of cortactin in the soma of the same neuron. To quantify synaptic CTTNBP2, we performed line scanning from the tip of the dendritic spine to the dendritic shaft. The CTTNBP2 signals along the lines were determined using the line scanning method in ImageJ (NIH). The signal within the range of 0-0.5 μm from the spine tip indicated the synaptic region, whereas signals within the range 1-1.5 μm from the tip represented the base of the dendritic spine and the dendritic shaft. Five spines of each neuron were randomly sampled blindly from thirty-five neurons for each group.

To quantify the acetyl-tubulin/tubulin ratio in neurons, we measured a 5-μm segment of primary dendrite within the range of 5–20 μm away from the soma, and used the α-tubulin signal to normalize the acetyl-tubulin signal. This ratio represents microtubule stability in proximal dendrites.

Detailed information on morphometry sampling (number of examined mice, neurons, dendrites, and spines) is presented in Additional file 2: Table S1.

### mEPSC recording

Cultured rat hippocampal neurons were transfected at 14 DIV, and whole-cell patch-clamps were performed at 18 DIV to record miniature EPSCs (mEPSCs). Neurons were incubated in extracellular solution containing 136.5 mM NaCl, 5.4 mM KCl, 1.8 mM CaCl_2_.2H_2_O, 0.53 mM MgCl_2_.6H_2_O, 5.56 mM Glucose, 5 mM HEPES (4-(2-hydroxyethyl)-1-piperazineethanesulfonic acid) pH7.4(NaOH), 0.001 mM tetrodotoxin, and 0.02 mM bicuculline. The intracellular solution contained 140 mM K-gluconate, 5 mM NaCl, 2 mM EGTA (ethylene glycol-bis(β-aminoethyl ether)-N,N,N′,N′-tetraacetic acid), 10 mM HEPES, 4 mM Mg-ATP, 0.3 mM Na-GTP pH 7.3(KOH). Neurons were voltage-clamped at −70 mV, and mEPSCs were recorded with an Axon Axopatch 200B amplifier (Molecular Devices) and filtered at 1 kHz. Clampfit software (10.4; Molecular Devices) was used to detect mEPSCs from the raw data with an amplitude threshold of 5 pA.

### Immunoprecipitation

The antibody-protein A complex was first prepared by incubating 20 μl of myc antibody (9B11, Cell Signaling) with 20 μl of Protein A beads (17046901, GE Healthcare) overnight and washing with PBS to remove unbound antibody. To prepare protein extract for cortactin/oligomerization coimmunoprecipitation experiments, COS1 cell lysates were extracted with RIPA buffer [1% Triton X-100, 0.1% sodium dodecyl sulfate (SDS), 1% sodium deoxycholate, 50 mM Tris–Cl pH 7.4, 150 mM NaCl, 2 mM EDTA and protease inhibitors] and the debris was removed by centrifugation (16,000 × *g* for 20 min at 4 °C using a table-top microcentrifuge, Heraeus Biofuge Fresco). Lysate was incubated with myc tag antibody-coated Protein A beads for 4 h at 4 °C and washed once with each of the following buffers: (1) RIPA buffer, (2) 10 mM Tris–Cl, 1% Triton  ×  100, pH7.4, (3) 10 mM Tris–Cl, 0.1% Triton  ×  100, 0.5 M LiCl, pH7.4, and (4) 10 mM Tris–Cl, pH7.4. For GFP-P-rich coimmunoprecipitation, lysates were extracted with 1% Triton  ×  100 in PBS and incubated with myc tag antibody-coated Protein A beads for 4 h at 4 °C and washed three times with PBS. After removing the final wash buffer, 2 ×  SDS-PAGE sample buffer (4% SDS, 0.2% (w/v) bromophenol blue, 20% (v/v) glycerol, 200 mM β-mercaptoethanol) was added and boiled for 10 min.

### Microtubule spin down assay

A Microtubule Binding Protein Spin-down Assay Biochem Kit (BK029, Cytoskeleton, Denver, CO) was used to test the interaction of CTTNBP2 with microtubule according to the manufacturer’s instructions. Tubulin solution was added into General Tubulin Buffer with Taxol (GTB; 80 mM PIPES pH 7, 2 mM MgCl_2_, 0.5 mM EGTA, 1 mM GTP and 20 mM Taxol) at 35 ˚C for 20 min to induce microtubule formation. To prepare CTTNBP2 proteins, COS1 cells were transfected with either WT or D570Y mutant expression plasmid. One day later, soluble total protein lysates containing CTTNBP2 proteins were collected from the supernatants after centrifugation at 16,000  ×  g for 20 min at 4 °C (table-top microcentrifuge, Heraeus Biofuge Fresco). Different amounts of protein lysates were incubated with or without microtubule at room temperature for 30 min. The samples were then placed onto 100 μl cushion buffer (60% glycerol in GTB) and centrifuged at 100,000  ×  g for 40 min at room temperature. The pellet fraction is the microtubule binding fraction and the supernatant is the unbound fraction. The sample was then boiled in 2 ×  sample buffer for 10 min and analyzed by immunoblotting.

### Mouse behavioral assays

#### Open field

The open field test was conducted as described previously [6, 12] to monitor locomotor activity and anxiety. Briefly, the test animal was placed in the center of a transparent acrylic box (40 × 40 × 30 cm) and allowed to freely explore the environment. The experiment was videotaped for 10 min from above the box. The central zone of the box was defined by a square (20 × 20 cm) equidistant from the walls. The area of the defined central zone is equal to the sum of the four corners. To track and analyze the movement of the mice, the Smart Video Tracking System (Panlab) was employed. Total moving distance (to indicate locomotor activity) and the ratio of time spent at the center to that at the corner (to represent the degree of anxiety) were measured.

#### Elevated plus maze

A plus maze composed of two open arms and two closed arms (30 × 5 cm) extending from a small central platform (5 × 5 cm) was elevated from the floor to a height of 45.5 cm for the test. The test mouse was placed at the center of the platform and allowed to freely explore the environment for 10 min. The Smart Video Tracking System (Panlab) was used to track the movement of the animal. The percentages of time spent in open arms and closed arms were assessed.

#### Reciprocal social interaction (RSI)

Test mice were individually housed for approximately one week before the experiment. A stranger adult male mouse of the same age as test mice or one week younger was placed into the home cage of the test mouse for 10 min. During the entire session, the lid of the cage was kept open to limit aggressive behaviors. Mouse behaviors were recorded by videotaping from above. The total time the test mouse spent sniffing the stranger mouse (head-to-head, head-to-body and head-to-anogenital) was manually recorded to represent social interaction.

#### Three-chambered test

The three-chamber was performed as described previously [10, 12]. In brief, the apparatus was a rectangular transparent plastic box (17.5 × 41.4 × 22 cm), with two dividing walls that separate the box into three equal chambers. Each dividing wall had a sliding entrance to connect different chambers. Two cylindrical wire cages (10.5 cm in diameter and 11 cm in height) were put in the left and right chambers. The experiment comprised three 10-min sessions (habituation, sociability and novelty preference), with 5 min intervals between each session. During intervals, test mice were placed back in their home cage. In every session, the test mouse was placed into the central chamber and the two sliding doors were then simultaneously opened to allow the mouse to freely explore the whole environment. In the habituation session, both cylindrical wire cages were empty. In the sociability test session, an object (Ob) was placed in one of the wire cages and a stranger mouse (S1) was placed in the other wire cage. In the social novelty preference session, the object was replaced by another stranger mouse (S2). Mouse behaviors were recorded by videotaping from above. Sniffing toward the cylindrical wire cages was considered as social interaction, which was quantified manually without knowing the genotype of mice. The value of (T_S1_-T_Ob_)/(T_S1_ + T_Ob_) was defined as the preference index of sociability. The value of (T_S2_-T_S1_)/(T_S2_ + T_S1_) was defined as novelty preference. T_Ob_ is the interaction time with the object, T_S1_ is the interaction time with S1, and T_S2_ is the interaction time with S2.

#### Statistical analyses

Statistical analysis and graphical outputs were performed using PRISM 5.03 or 8.3 (Graphpad software). All sample sizes, statistical data and corresponding statistical methods are summarized in Tables S1 and S2. In brief, to compare multiple groups with one variant, one-way ANOVA with Bonferroni multiple comparison post hoc test was performed for normally distributed data and Kruskal–Wallis test with Dunn’s multiple comparison test was performed for nonparametric distributed data. To compare multiple groups with two variants, two-way ANOVA was employed. To compare two groups of unrelated datasets, two-tailed Mann–Whitney test or unpaired t test was employed. In this report, *P *< 0.05 is regarded as significant. For cumulative distribution analysis, a Kolmogorov–Smirnov (K-S) test was performed (https://www.aatbio.com/tools/kolmogorov–smirnov-k-s-test-calculator). Outliers in the dataset were excluded using the “Identify outlier” tool in PRISM. All image analyses, including immunoblotting and morphometric analyses, were conducted using ImageJ (NIH). All morphometric and behavioral assay data were analyzed blind to minimize personal bias by relabeling the samples before being analyzed by another member of the laboratory. All statistical methods and results are summarized in Additional file 2: Table S1, Additional file 3: Table S2.

## Results

### ASD-linked mutations alter dendritic spine formation and the subcellular distribution of CTTNBP2

The ASD-linked mutations of *CTTNBP2* we targeted are distributed along the entire short form of the protein (Fig. 1a, upper). Since human, mouse and rat CTTNBP2 proteins are highly conserved and the residues of ASD-linked mutations are identical among these three species (Additional file 1: Fig. S1), we used both rat and mouse models to investigate seven ASD-linked mutations of *CTTNBP2*. Monoallelic mutation of CTTNBP2 has been identified in patients. Thus, we overexpressed *Cttnbp2* ASD-linked mutants in cultured neurons and used heterozygous knockin mutant mice to mimic the genetic condition of patients. The corresponding residues in mouse CTTNBP2 of the human mutations are R42W, A112T, M120I, G342R, P353A, R533* and D570Y (Fig. 1a, upper). Among these mutations, R533* mutation generated a truncated variant that lacks the C-terminal proline-rich domain. We expected R533* mutation would disrupt the interaction between CTTNBP2 and cortactin, impairing dendritic spine formation. For the remaining six mutations, there was no obvious clue to predict the impact of these mutations.

To characterize CTTNBP2, first we analyzed CTTNBP2 structure using IUPred2 (https://iupred2a.elte.hu/). Our results indicate that CTTNBP2 is an intrinsically unstructured molecule, apart from the N-terminal coiled-coil region (Fig. 1a, lower). Next, we expressed the seven ASD-linked mutations of mouse *Cttnbp2* in COS1 cells to confirm their expression (Fig. 1b). Since CTTNBP2 is critical for dendritic spine formation and maintenance [5, 20], we then investigated if these mutations alter dendritic spine formation. To do that, we individually transfected the seven ASD-linked mutant proteins into wild-type (WT) cultured hippocampal neurons. Overexpressed WT CTTNBP2 formed puncta at dendritic spines and presented a filamentous pattern along axons of mature cultured neurons at 18 days in vitro (DIV) (Fig. 1c). Similar to WT CTTNBP2, the R42W, A112T, G342R and P353A mutant proteins were enriched at dendritic spines (Fig. 1c). However, the M120I and R533* mutant proteins were evenly distributed throughout neurons (Fig. 1c). Moreover, the D570Y mutant protein formed filamentous bundles along dendritic shafts and particularly along the proximal dendrites, a phenotype that differed from WT as well as those of the other mutants (Fig. 1c).

Next, we quantified dendritic spine density to evaluate the impact of ASD-linked mutations on excitatory synapse formation (Fig. 1c). Coexpression of GFP-actin was performed to outline neuronal morphology, including dendritic spines. We found that overexpressing WT CTTNBP2 slightly increased dendritic spine density relative to vector control. Compared to WT, expression of the R42W, M120I, G342R and R533* mutants reduced dendritic spine density in cultured neurons. In particular, the dendritic spine densities of the M120I and R533* mutant neurons were even lower than for neurons transfected with vector control (Fig. 1c). Thus, R42W and G342R might represent loss-of-function mutations that do not exert the same beneficial effect on spinogenesis as WT protein, whereas M120I and R533* may have dominant-negative effects that disrupt the function of endogenous CTTNBP2 (Fig. 1c, Table S1. Note that all statistical results in this report are summarized in Additional file 2: Table S1, Additional file 3: Table S2).

To further corroborate the effect of M120I and R533* on dendritic spine density, we performed patch clamp recording to measure the miniature excitatory postsynaptic current (mEPSC) of M120I or R533* mutant protein-expressing neurons. Compared with WT, expression of M120I or R533* mutant proteins reduced the frequency, but not amplitude, of mEPSCs (Fig. 1d), echoing the effect of M120I or R533* mutant proteins on dendritic spine formation. Though expression of the D570Y mutant protein did not alter dendritic spine density, we were intrigued by its unique filamentous pattern along dendritic shafts. Hereafter, we focus on further dissecting how the M120I, R533* and D570Y mutations alter CTTNBP2 distribution and function.

### M120I and R533* mutant proteins display reduced interactions with cortactin

**Fig. 2:**
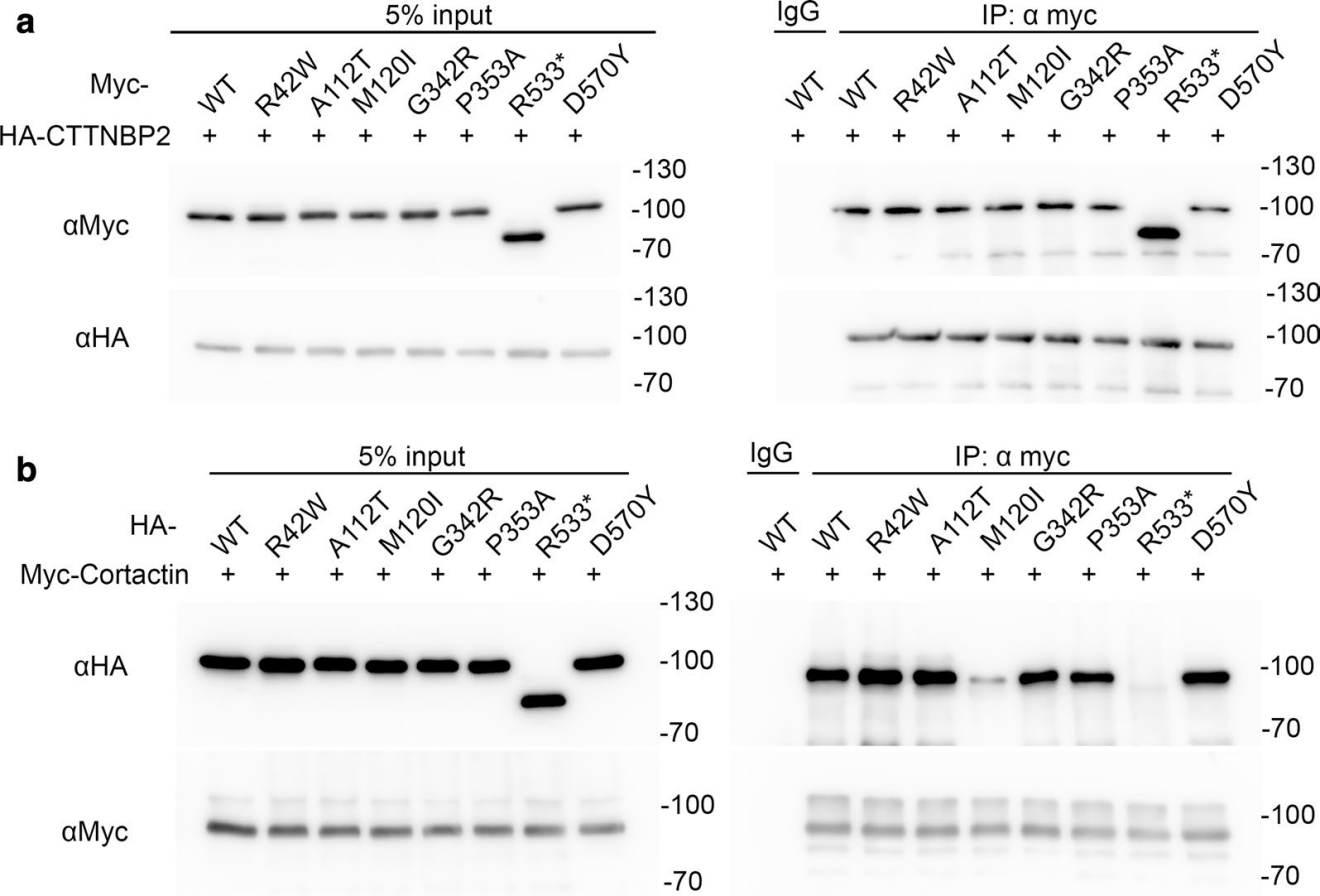
M120I and R533* mutations impair the C-terminal protein interactions of CTTNBP2. Co-immunoprecipitation (IP) using transfected COS1 cell lysates was performed to analyze the protein–protein interactions of seven ASD-linked mutations of CTTNBP2. **a** The interactions between HA-tagged WT CTTNBP2 and Myc-tagged ASD mutants. **b** The interaction between Myc-tagged cortactin and various HA-tagged CTTNBP2 constructs from transfected COS1 cells. Myc tag mouse antibody was used for IP, and both Myc and HA antibodies (from rabbit and rat, respectively) were used for immunoblotting, as indicated

Since CTTNBP2 acts as a synaptic scaffold protein, we applied biochemical approaches to analyze the protein–protein interactions of *Cttnbp2* mutants. The M120I mutation is located in the N-terminal coiled-coil domain, which is involved in CTTNBP2 hetero- and homo-oligomerization [4, 21]. Both the R533* and D570Y mutations occur in the C-terminal P-rich region, which is required for cortactin interaction [5]. We used transfected COS1 cells to investigate if these mutations influence the interactions of CTTNBP2 with its binding partners, including cortactin and CTTNBP2 itself. We found that all Myc-tagged WT and the ASD-linked CTTNBP2 mutant proteins were able to co-immunoprecipitate with WT CTTNBP2 from COS1 lysates (Fig. 2a), suggesting that ASD-linked mutations do not alter CTTNBP2 oligomerization. To further evaluate the interaction between CTTNBP2 and cortactin, we cotransfected Myc-tagged cortactin and various HA-tagged CTTNBP2 constructs into COS1 cells. Myc-tag antibody was then used to immunoprecipitate cortactin. As anticipated, R533* mutant protein was not present in the precipitate, indicating that R533* mutation indeed disrupts the interaction between CTTNBP2 and cortactin. However, unexpectedly, we found that the M120I mutant protein exhibited greatly reduced interaction with cortactin (Fig. 2b), indicating that this N-terminal M120I mutation influences the C-terminal structure or its accessibility for cortactin interaction.

### Cortactin overexpression ameliorates dendritic spine deficits caused by M120I and R533* mutations

**Fig. 3:**
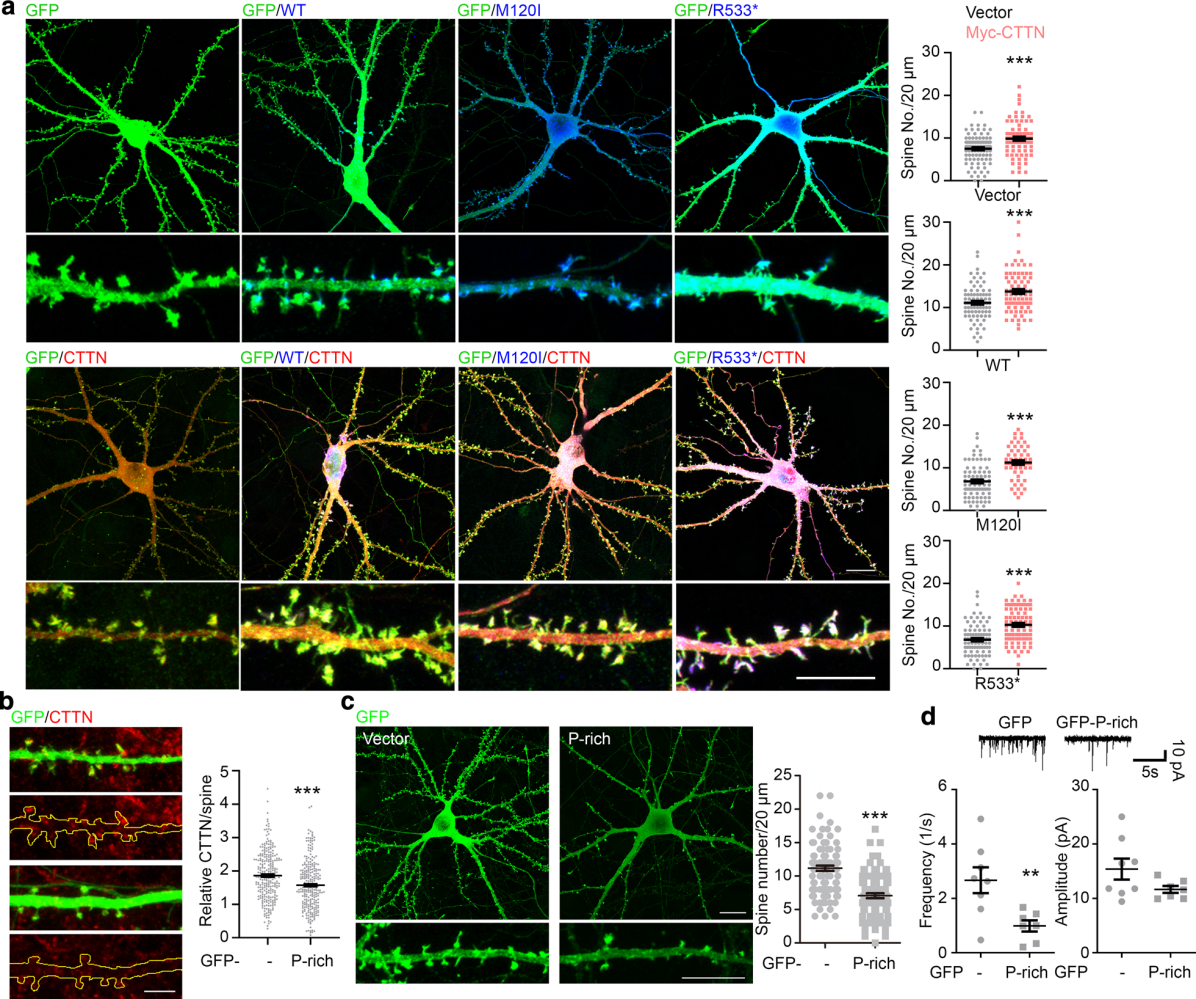
Interaction with cortactin via the C-terminal P-rich domain of CTTNBP2 is essential for dendritic spine formation and activity. **a** Cortactin overexpression rescues dendritic spine density in neurons expressing M120I or R533* mutant protein. HA-tagged WT, M120I or R533* CTTNBP2 was cotransfected with Myc-tagged cortactin (CTTN) and GFP-actin. In M120I- and R533*-transfected neurons, cortactin expression rescues dendritic spine density. Quantification results of dendritic spine density are shown. **b** Overexpression of the GFP-P-rich construct reduced the synaptic distribution of cortactin in mature cultured hippocampal neurons. The signal of cortactin in individual spines was normalized with the signal for cortactin in the soma to represent synaptic enrichment of cortactin. **c** Overexpression of the GFP-P-rich construct reduces dendritic spine density. **d** GFP-P-rich overexpression reduces mEPSC frequency, but not amplitude. GFP was used as a control in (**b**)–(**d**). Data represent mean ± SEM from three independent experiments. Each dot in (**a**, **c**), (**b**) and (**d**) represents an individual dendrite, spine or, neuron, respectively. Two-tailed unpaired *t* tests were performed. All statistical data and exact sample sizes are available in Additional file 2: Table S1. *P < 0.05; **P < 0.01; ***P < 0.001; ns, not significant. Scale bar: **a** upper, 20 μm; lower, 10 μm; **b** 5 μm; **c** upper, 20 μm; lower, 10 μm

Our previous report indicated that cortactin is a critical downstream factor of CTTNBP2 to control dendritic spine formation. Cortactin overexpression rescues dendritic spine defects caused by *Cttnbp2* knockdown [5]. Since the M120I and R533* mutant proteins are both defective in their interaction with cortactin, we hypothesized that cortactin overexpression would rescue the dendritic spine defects caused by these two mutations. To test that possibility, we triply transfected GFP-actin, HA-tagged CTTNBP2 variants (including WT, M120I and R533*) and Myc-tagged cortactin or vector control into cultured hippocampal neurons (Fig. 3a). Indeed, cortactin overexpression increased the dendritic spine density of M120I- or R533*-expressing neurons (Fig. 3a), evidencing an important role for cortactin-mediated dendritic spine formation in the ASD pathogenic pathway induced by CTTNBP2 mutations.

### The P-rich domain of CTTNBP2 is critical for controlling dendritic spine density and synaptic activity

To further validate the significance of the interaction between cortactin and CTTNBP2 for dendritic spine formation, we overexpressed the GFP-tagged P-rich (GFP-P-rich) domain of CTTNBP2 to disrupt the interaction between endogenous CTTNBP2 and cortactin in cultured neurons. We found that GFP-P-rich overexpression reduced the synaptic distribution of cortactin (Fig. 3b). Consistent with the essential role of cortactin in dendritic spine formation, GFP-P-rich overexpression also resulted in decreased dendritic spine density (Fig. 3c). Moreover, measurement of mEPSCs revealed a reduction in frequency but not amplitude due to GFP-P-rich overexpression (Fig. 3d). These findings support that the interaction between cortactin and the C-terminal P-rich domain of CTTNBP2 is fundamental to dendritic spine formation and synaptic activity.

### M120I mutation disrupts the N–C terminal interaction of CTTNBP2

**Fig. 4:**
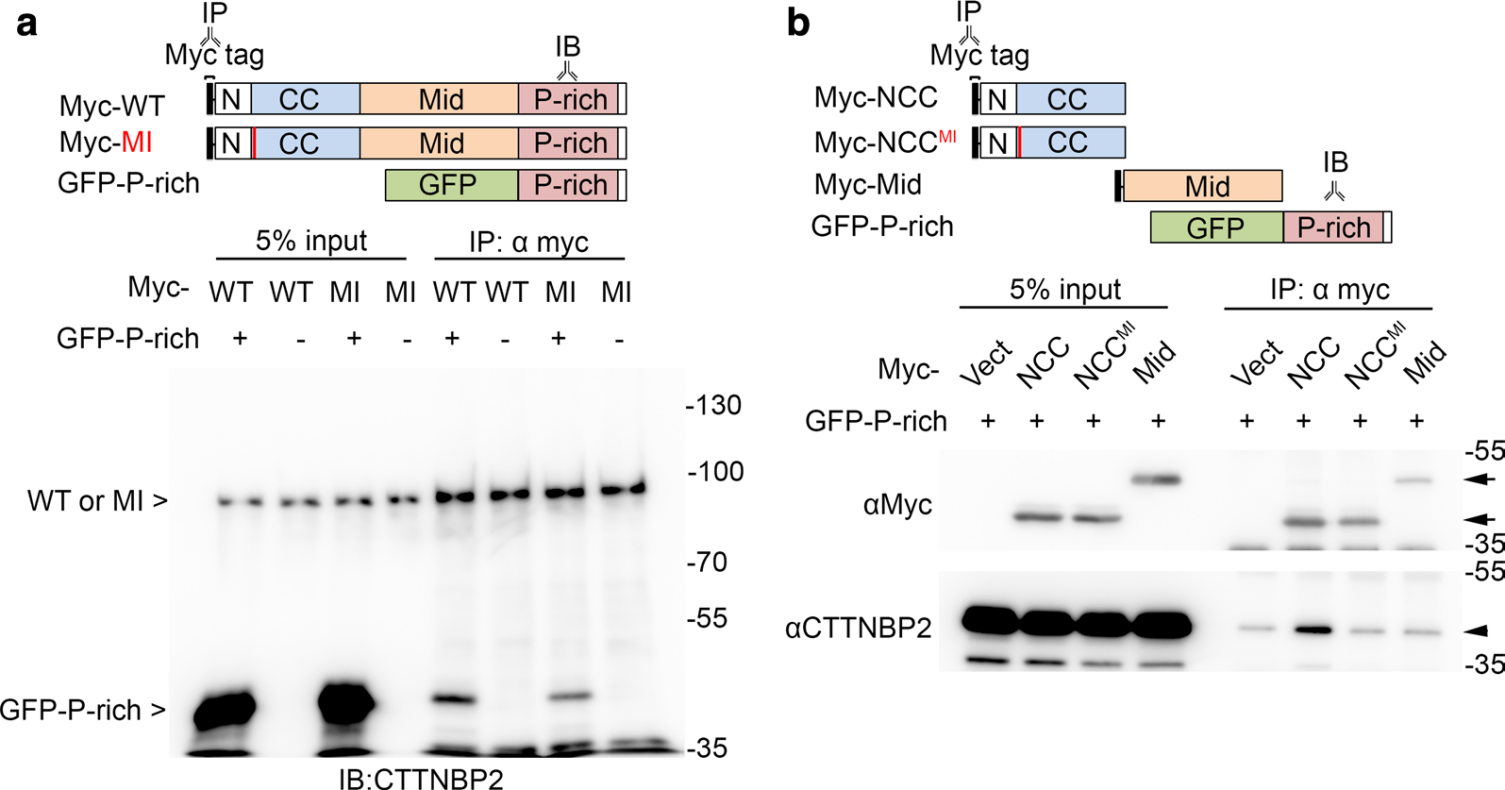
M120I mutation impairs the N–C terminal interaction of CTTNBP2. **a** M120I mutation compromises the association of full-length CTTNBP2 with GFP-P-rich fragments. Top: schematics of constructs. Bottom: co-immunoprecipiation (IP) results. Full-length Myc-tagged wild type (Myc-WT) or M120I (Myc-MI) CTTNBP2 proteins were precipitated using Myc tag antibody. Antibody recognizing the C-terminal CTTNBP2 region was used in immunoblotting to recognize all Myc-WT, Myc-MI and GFP-P-rich constructs. GFP vector was used as negative control. **b** M120I mutation compromised the association of GFP-P-rich fragments with the NCC fragment of CTTNBP2. Top: schematics of constructs. Bottom: co-immunoprecipitation results. Myc-tagged NCC domains (either wild type Myc-NCC or M120I-mutant Myc-NCC^MI^) were precipitated by Myc tag antibody. Vector only or Mid domain was used as a negative control. Myc and CTTNBP2 antibodies were used for immunoblotting. Arrows point to Myc-tagged constructs, whereas arrowhead indicates GFP-P-rich construct

The M120 residue is located at the beginning of the coiled-coil (CC) domain, a region far from the C-terminal cortactin binding region (Fig. 1a). We were surprised to find that the M120I mutation also diminished the CTTNBP2-cortactin interaction. Since CTTNBP2 is an intrinsically unstructured molecule (Fig. 1a), it implies that CTTNBP2 proteins are inherently flexible. Accordingly, we postulated that a N–C terminal interaction modulates the C-terminal protein–protein interactions of CTTNBP2. To validate the N–C interaction, we cotransfected full-length Myc-tagged WT or M120I mutant protein with GFP-P-rich fragments into COS1 cells and conducted immunoprecipitation with Myc tag antibody (Fig. 4a). Indeed, GFP-P-rich fragments co-immunoprecipitated with WT CTTNBP2, whereas association of the M120I mutant with GFP-P-rich fragments was reduced relative to WT (Fig. 4a). This outcome indicates that the M120I mutation interfered with the N–C interaction. To further confirm this point, we co-transfected Myc-tagged WT N-terminal coiled-coil domain (NCC^WT^) or the NCC domain with the M120I mutation (NCC^MI^) together with GFP-P-rich fragments. We observed a much stronger interaction of NCC^WT^ with GFP-P-rich fragments than NCC^MI^ (Fig. 4b). The interaction was specific because the Myc-tagged Mid domain did not have the same ability to precipitate the GFP-P-rich fragments (Fig. 4b). These results indicate that the N-terminal coiled-coil domain of CTTNBP2 interacts with the protein’s C-terminal P-rich domain, and that this interaction is compromised by M120I mutation.

Based on these analyses, we suggest that the impaired dendritic spine formation caused by the M120I and R533* mutant proteins is due to their incapacity to interact with cortactin. However, different mechanisms are responsible for disrupting the interaction between these two mutant CTTNBP2 proteins and cortactin.

### D570Y mutation enhances the microtubule association of CTTNBP2 and microtubule stability

**Fig. 5:**
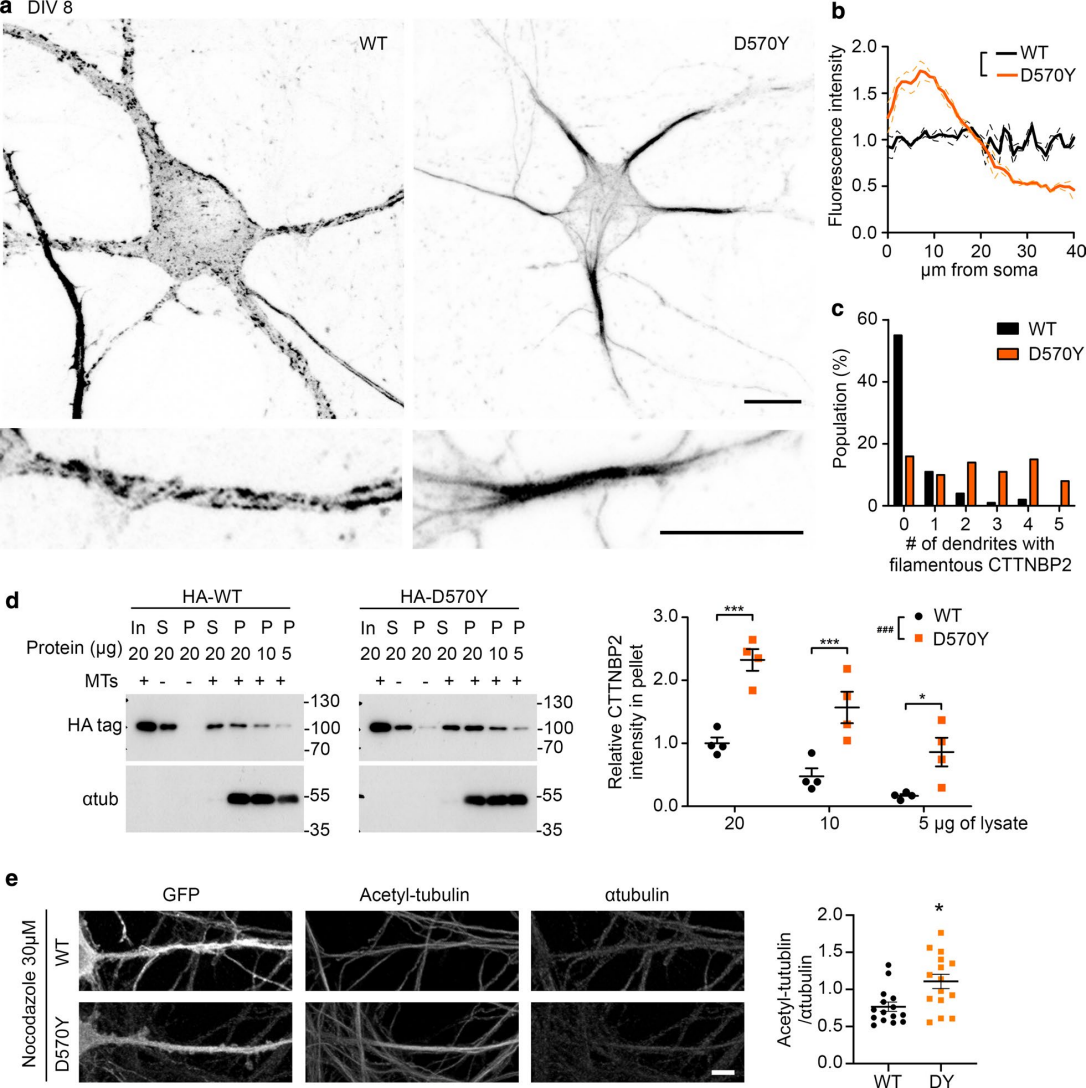
D570Y mutation alters the subcellular distribution and microtubule association of CTTNBP2. **a** Peculiar distribution of CTTNBP2 D570Y mutant protein in cultured hippocampal neurons at DIV 8. **b** Average immunoreactivities of WT and D570Y mutant protein along dendrites. **c** D570Y-expressing neurons possess more dendrites expressing filamentous CTTNBP2. **d** Increased microtubule association of CTTNBP2 upon D570Y mutation. COS1 cells were separately transfected with WT or D570Y CTTNBP2 and subjected to microtubule spin down assay using microtubule filaments. Immunoblotting using HA tag and α-tubulin antibodies was then performed to analyze the results. Quantification results of four independent experiments are shown in the panel at right. **e** Increased tubulin acetylation upon expression of D570Y mutant. Cultured hippocampal neurons were cotransfected with GFP and WT or D570Y mutant CTTNBP2. After nocodazole treatment, neurons were subjected to immunostaining using antibody against acetylated tubulin. Normalized signals of acetylated tubulin are shown. Data in (**b**), (**d**) and (**e**) represent mean ± SEM. In (**d**), each dot indicates an independent immunoblotting experiment. In (**e**), one dot represents the average result of one neuron. Data in (**c**) represent the data collected from three independent experiments. WT, n = 73, D570Y, n = 74. (**b**), (**d**) Two-way ANOVA; (**e**) two-tailed Mann–Whitney test. All statistical data and exact sample sizes are available in Additional file 2: Table S1. *P < 0.05; **P < 0.01; ***P < 0.001; ns, not significant. Scale bar: **a** 10 μm; **e** 5 μm

We then investigated if and how the D570Y mutation influences CTTNBP2 function. Since CTTNBP2 also associates with microtubule and distributes along the dendritic shaft mainly before mature spine formation [21], the distribution of D570Y mutant proteins at the dendritic shaft of mature neurons (Fig. 1c) suggests a potential alteration of the interaction between microtubule and CTTNBP2 protein. To investigate that possibility, we first investigated the distribution of D570Y mutant protein in immature hippocampal neurons. Similar to our previous finding [21], WT CTTNBP2 formed punctate aggregates along dendrites at DIV 8 (Fig. 5a, left). However, D570Y mutant protein preferentially formed filamentous structures and exhibited a peculiar accumulation in proximal dendrites (Fig. 5a). We quantified the intensity of CTTNBP2 signal along dendrites and found that, unlike WT protein, D570Y mutant protein strongly accumulated along dendrites within a distance of 20 μm from the soma (Fig. 5b). Furthermore, the D570Y mutant-expressing neurons tended to have multiple dendrites with CTTNBP2 filaments (Fig. 5a, c). Thus, our results indicate that the D570Y mutation alters the subcellular distribution of CTTNBP2 in neurons.

To investigate if D570Y mutation results in a higher affinity to bind microtubule, we performed a microtubule binding protein spin-down assay. COS1 lysates that expressed WT or D570Y mutant CTTNBP2 proteins were mixed with preassembled microtubule filaments and subjected to centrifugation to examine if microtubule can bring down more D570Y mutant protein relative to WT. By applying three different lysate amounts (i.e. 5, 10 and 20 μg of protein) in our microtubule spin-down assay, we found that both WT and D570Y mutant protein exhibited a dosage-dependent microtubule association, but amounts of microtubule binding by D570Y mutant protein were 2.5-4-fold higher than those displayed by WT protein (Fig. 5d).

Given the higher affinity for microtubule of D570Y mutant protein relative to WT, it is likely to increase microtubule stability. To monitor microtubule stability in neurons, we transfected WT or D570Y mutant protein into cultured hippocampal neurons and added nocodazole to disrupt the microtubule network. Then, we analyzed the level of acetylated tubulin, a stable tubulin marker, within the proximal dendrites of our cultured hippocampal neurons. Compared with neurons transfected with WT CTTNBP2, neurons transfected with D570Y mutant protein displayed higher levels of acetylated tubulin within proximal dendrites (Fig. 5e), supporting that the D570Y mutation increases microtubule stability.

Thus, our experiments have shown that D570Y mutant protein has a higher affinity for microtubule, resulting in increased microtubule stability.

### D570Y mutation reduces the dendritic spine distribution of CTTNBP2 and dendritic spine density

**Fig. 6:**
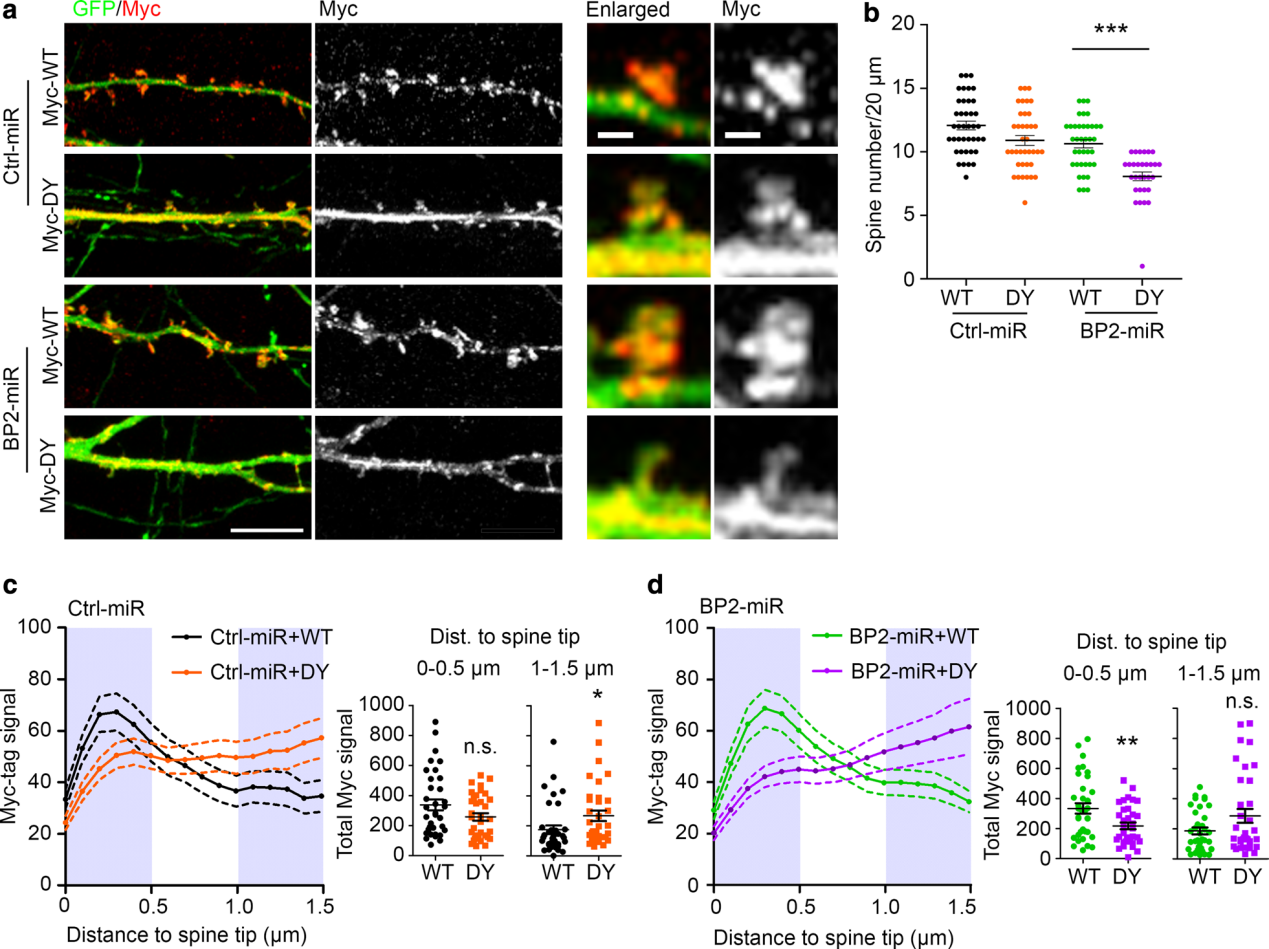
D570Y mutation reduces the synaptic distribution of CTTNBP2 and impairs dendritic spine formation in *Cttnbp2* knockdown neurons. **a** D570Y (DY) mutation impairs synaptic targeting of CTTNBP2. Expression pattern of WT and D570Y mutant protein in *Cttnbp2* knockdown (BP2-miR) and control (Ctrl-miR) hippocampal neurons. Representative images of dendrites and enlarged spines are shown. **b** Overexpression of D570Y mutant protein cannot rescue the density of dendritic spines in miR-BP2 neurons. (**c**)–(**d**) Reduced Myc-D570Y signal at the tips of dendritic spines. The results of line scanning to measure the distribution of Myc-tagged WT and D570Y mutant proteins from the tip to the base of dendritic spines in Ctrl-miR- **c** and BP2-miR-expressing (**d**) neurons. Left: average intensity along dendritic spines. Right: sums of the signal between 0 and 0.5 or 1 and 1.5 μm from spine tip (shaded in grey in left panels) of each neuron. Data represent mean ± SEM. Each dot in (**b**, **c**, **d**) indicates the individual result of a neuron. All results were randomly collected in a blind fashion from three independent experiments. Data in (**b**) were analyzed by two-way ANOVA with Bonferroni’s multiple comparisons test. Data in (**c**) and (**d**) were analyzed with a Mann–Whitney test. All statistical data and exact sample sizes are available in Additional file 2: Table S1, Additional file 3: Table S2. *P < 0.05; **P < 0.01; ***P < 0.001; ns, not significant. Scale bar: **a** left, 10 μm; right, 1 μm

Since D570Y mutant protein displayed greater ability to bind microtubule and given that microtubule is mainly present in the dendritic shaft but not dendritic spines, we expected that the D570Y mutation would result in CTTNBP2 being retained in the dendritic shaft, thereby reducing the synaptic distribution of CTTNBP2 in mature neurons. However, our data shown in Fig. 1c suggest that the D570Y mutation does not overly perturb dendritic spine formation. Potentially, self-oligomerization with endogenous WT CTTNBP2 enables exogenous D570Y mutant protein to enter dendritic spines and maintain dendritic spine formation. If this speculation is correct, a reduction of endogenous WT CTTNBP2 should result in impaired dendritic spine distribution of D570Y mutant protein. To investigate that possibility, we applied the previously-established knockdown construct BP2-miR to reduce endogenous WT CTTNBP2 protein [5]. BP2-miR-resistant WT and D570Y mutant proteins were then cotransfected with BP2-miR or control vector Ctrl-miR into neurons (Fig. 6a). We found that compared to WT CTTNBP2, expression of the D570Y mutant protein indeed resulted in a reduced dendritic spine density in BP2-miR-expressing neurons (Fig. 6b). This finding is consistent with our speculation that endogenous WT CTTNBP2 neutralizes the impact of the D570Y mutation.

Next, we applied line-scanning from the tip to the base of dendritic spines to quantify the intensity of WT and D570Y mutant proteins in dendritic spines (Fig. 6c, d). Immunoreactivity of WT CTTNBP2 was enriched at the tips of dendritic spines (within 0-0.5 μm from the tip) and declined as distance from the tip increased, particularly within the region of 1-1.5 μm from the tip in both BP2-miR- and Ctrl-miR-expressing neurons (Fig. 6c, d). In contrast, we noted a high level of D570Y mutant protein expression within the 1-1.5 μm region (Fig. 6c, d). In fact, signal of D570Y mutant protein was much lower around the tips of dendritic spines from BP2-miR-expressing neurons (Fig. 6d). In Ctrl-miR-expressing neurons, synaptic distributions of WT and D570Y mutant protein were comparable (Fig. 6c). Together, these results indicate that the D570Y mutation indeed reduces synaptic targeting of CTTNBP2.

### D570Y and M120I mutations result in dendritic spine deficits in mice

**Fig. 7:**
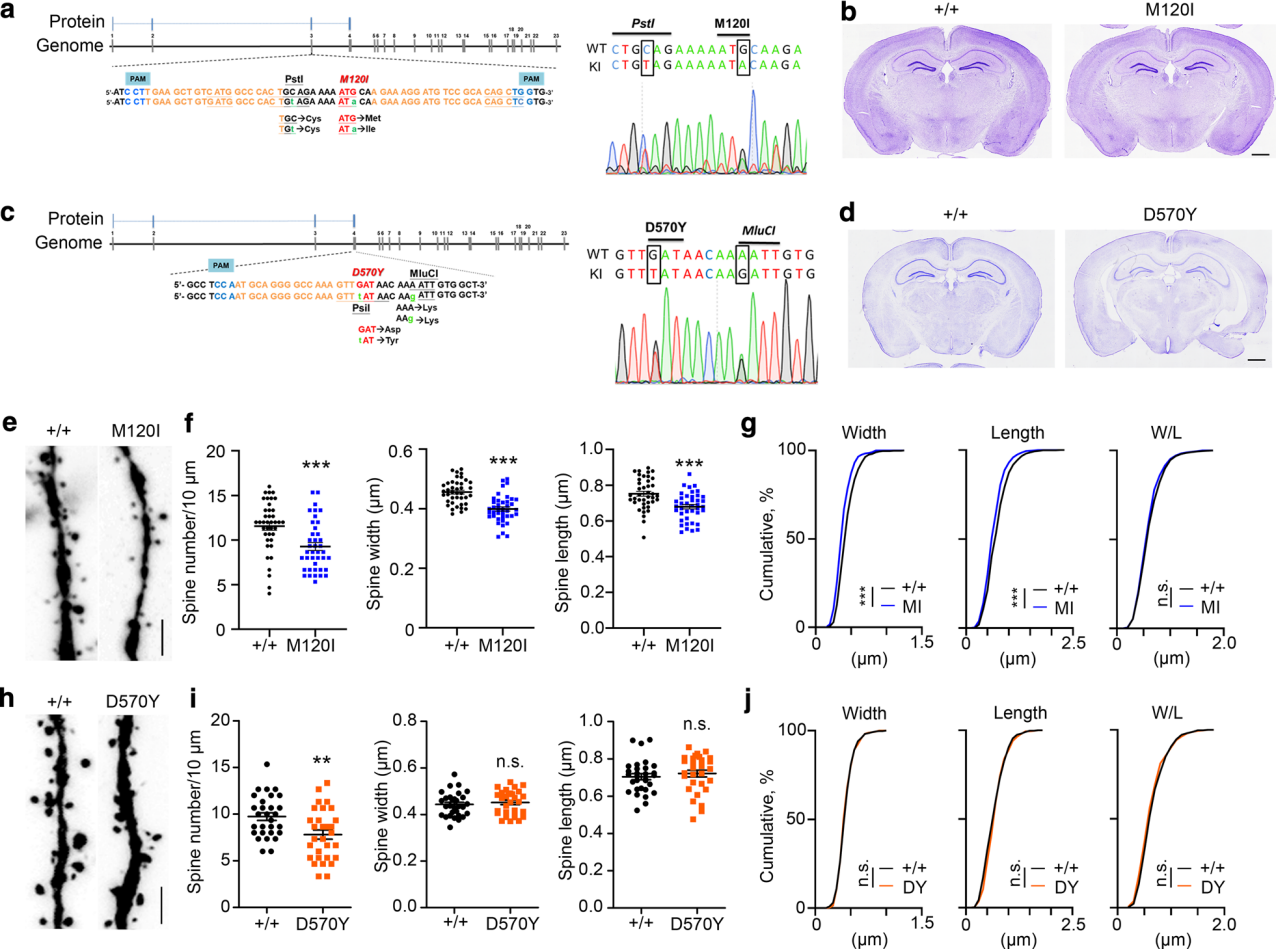
M120I and D570Y mutations of CTTNBP2 reduce dendritic spine density in vivo. **a**, **c** Design and mutation sites of *Cttnbp2* M120I and D570Y knockin mice using CRISPR/Cas9 editing technology as indicated. Sequencing results of heterozygous mice are shown. **b**, **d** Cresyl violet staining reveals no obvious brain anatomical defect of *Cttnbp2* M120I and D570Y knockin heterozygous mice. **e**–**j** Both M120I and D570Y mutations reduce dendritic spine density and only the M120I mutant alters dendritic spine morphology. Dendritic spine analysis of M120I (**e**–**g**) or D570Y (**h**–**j**) mutant mice based on the signals of Thy1-YFP. **e**, **h** Representative images of the first dendritic branches of CA1 neurons and quantification results are shown. **f**, **i** Quantification of dendritic spine density and morphology. Data represent mean ± SEM and the results of individual neurons are also shown. For (f), N = 4 mice and n = 40 neurons. For (**i**), N = 3 mice and n = 30 neurons. **g**, **j** Cumulative probability of individual spine morphology in M120I (MI) or D570Y (DY) mice as indicated. For M120I spine density and D570Y spine width and length, two-tailed Mann–Whitney tests were performed. For M120I spine width and length and D570Y spine density, two-tailed unpaired *t* tests were performed. For cumulative probabilities, Kolmogorov–Smirnov (K–S) tests were performed. All statistical data and exact sample sizes are available in Additional file 2: Table S1. *P < 0.05; **P < 0.01; ***P < 0.001; ns, not significant. Scale bar: (**b**, **d**) 1 mm; (**e**, **h**) 2.5 μm

To further investigate the physiological relevance of ASD-linked mutations of *Cttnbp2*, we employed CRISPR/Cas9 genomic editing technology to generate M120I, R533* and D570Y knockin mice (Fig. 7a–d). Results for R533* mice have been reported previously [20]. Since only one of the two *CTTNBP2* alleles in ASD patients carries one of these three mutations, we used heterozygous KI mice to mimic patient conditions. Hereafter, M120I and D570Y mutant mice denote these heterozygous knockin mice. Note, to avoid potential off-target effects, all mutant mice were backcrossed to WT mice for more than six generations before undergoing experimentation.

Similar to *Cttnbp2* deletion and R533* mutant mice [20], neither M120I nor D570Y mutant mice displayed obvious anatomical defects in their brains (Fig. 7b, d). To investigate the dendritic spine features of these mutant mice, we crossed the mice with Thy1-YFP-H transgenic mice to outline neuronal morphology of forebrain projection neurons. Similar to our in vitro results, M120I mutant mice exhibited reduced density, width and length of dendritic spines in dorsal hippocampal CA1 neurons (Fig. 7e, f). We also compared the ratio of the width to the length (W/L) of individual spines and did not observe a statistical difference between M120I and WT neurons (Fig. 7g), suggesting a proportional shrinkage of dendritic spines in M120I mutant neurons. For D570Y mutant mice, although spine width and length were not altered (Fig. 7h–j), dendritic spine density was reduced compared to that of WT littermates (Fig. 7h–i). Thus, these results confirm that, similar to *Cttnbp2* deletion and R533* mutation, the M120I and D570Y mutations impair dendritic spine formation in vivo.

### M120I mutant mice exhibit impaired social interactions and neuronal activation

**Fig. 8:**
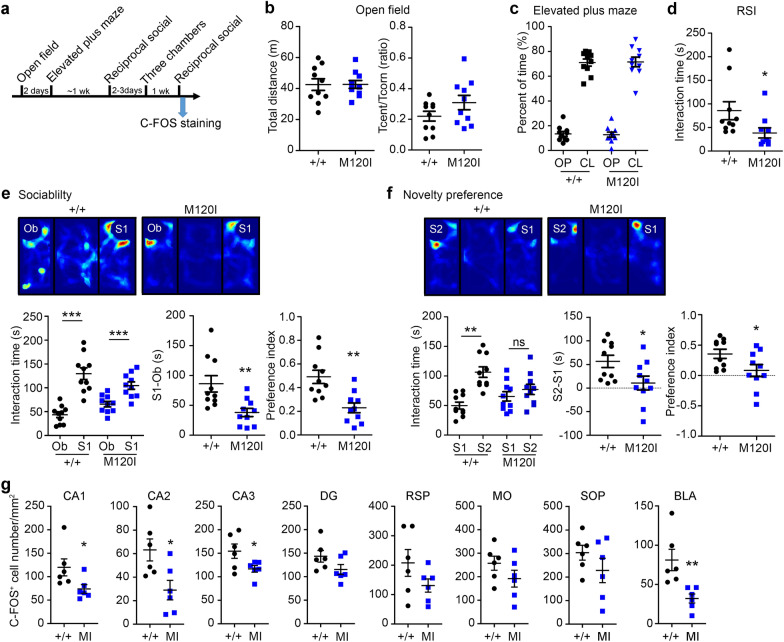
*Cttnbp2* M120I mutant mice exhibit reduced social interaction and neuronal activation. **a** Flowchart of behavioral assays used in this report. Two hours after the last assay, mouse brains were fixed and subjected to C-FOS staining. **b** Open field. **c** Elevated plus maze. **d** Reduced reciprocal social interaction (RSI) in M120I mutant mice. **e** Reduced sociability of M120I mutant mice in three-chamber test. **f** Reduced novelty preference of M120I mutant mice in three-chamber test. Heat maps of movement paths of mice, actual interaction time, the difference of interaction time between the left and right chambers, and preference index are shown. **g** C-FOS staining of M120I mutant mice after social stimulation. *DG* dentate gyrus, *RSP* retrosplenial cortex, *MO* motor cortex, *SOP* somatosensory cortex, *BLA* basal lateral amygdala. Data represent mean ± SEM and the results of individual mice are shown. **d** Two-tailed Mann–Whitney test. **e**–**f** A paired *t* test was used to compare total interaction times for the object (Ob) and stranger 1 (S1) or for S1 and stranger 2 (S2). To compare interaction times or preference indexes, an unpaired *t*-test or Mann–Whitney test was used. For (**g**), an unpaired *t*-test or Mann–Whitney test was used. All statistical data and exact sample sizes are available in Additional file 2: Table S1. *, *p *< 0.05; **, *p *< 0.01; ***, *p *< 0.001; ns, not significant

Recently, we reported that *Cttnbp2*–*/*–*, *+/– and R533* mutant mice all exhibit impaired social interaction behaviors [20], supporting a role for CTTNBP2 in regulating social behaviors. Here, we further assessed M120I mutant mice as a model to investigate if reduced social interaction is a typical trait induced by ASD-linked mutations in the *CTTNBP2* gene. We employed open field, elevated plus maze, reciprocal social interaction (RSI) and three-chamber tests to analyze M120I mutant mice (Fig. 8a). Similar to our report on R533* mutant mice [20], M120I mutant mice did not exhibit defects in their responses to open field and elevated plus maze tests (Fig. 8b, c), suggesting that M120I mutation does not influence locomotor activity or anxiety. For RSI, M120I mutant mice spent less time approaching the stranger mouse compared to wild-type littermates (Fig. 8d). In the sociability session of three-chamber test, although M120I mutant mice spent more time interacting with the stranger mouse (S1) rather than the object (Ob), both the time difference (i.e. S1–O) and the corresponding preference index of M120I mutant mice were lower than that of WT littermates (Fig. 8e). In the novelty preference session, wild-type littermates spent more time interacting with stranger mouse 2 (S2), but M120I mutant mice did not exhibit a preference for either S1 or S2 (Fig. 8f). These behavioral assays support that M120I mutation indeed results in reduced social behaviors and impaired social novelty preference, representing a core symptom of ASD.

Finally, we investigated if the behavioral deficits of M120I mutant mice are relevant to impaired neuronal activation. Two hours after our RSI test (Fig. 8a), we performed C-FOS staining on the test mice to monitor neuronal activation upon social stimulation. Similar to *Cttnbp2*^−*/*−^ mice [20], we found that neuronal activity of M120I mutant mice was lower at the CA1, CA2 and CA3 regions of hippocampus and basolateral amygdala compared to WT littermates (Fig. 8g).

In conclusion, together with our recently published results on R533* mutant mice [20], the findings presented in the current study suggest that ASD-associated *Cttnbp2* mutations indeed reduce dendritic spine density and neuronal activation and impair social behaviors in mice.

## Discussion

In this report, we collectively studied seven ASD-linked mutations of *Cttnbp2* from the aspects of biochemistry, cell biology and physiology. Based on our analyses, we characterized the effect of three potential disease-causative mutations, i.e. M120I, R533* and D570Y. These three mutations resulted in prominent dendritic spine defects in cultured hippocampal neurons, as well as in mouse brains. However, the mechanisms underlying the effects of these three mutations on dendritic spine formation differ, representing an illustrative example of how divergent mechanisms contribute to ASD etiology, even for mutations of the same gene, yet result in convergent outcomes.

Both the M120I and R533* mutations impair interactions of CTTNBP2 with cortactin, but the underlying mechanisms differ. R533* mutant protein lacks the C-terminal P-rich domain of CTTNBP2, so it lacks the ability to interact with cortactin. We found that overexpression of cortactin rescues the dendritic spine defects caused by R533* mutation, confirming a critical role for cortactin in the CTTNBP2 pathway. Since CTTNBP2 acts as a synaptic scaffold to regulate synaptic targeting of other proteins, such as SHANKs, NMDAR and protein phosphatase 2A regulatory subunit STRN [20], it is likely that the downstream effectors of CTTNBP2 and cortactin also crosstalk to other CTTNBP2-regulated proteins and consequently control dendritic spine formation.

The M120I mutation disrupts the N–C terminal interaction of CTTNBP2 proteins, impeding cortactin interaction. Cortactin overexpression also rescued the dendritic spine deficits caused by M120I mutation, further confirming the involvement of cortactin in how CTTNBP2 controls dendritic spine formation and neuronal function. Certainly, it is possible that the disrupted N–C interaction also influences other protein–protein interactions of CTTNBP2 and impairs coordination of cortactin and other CTTNBP2-interacting proteins. Apart from its N-terminal CC domain, CTTNBP2 is intrinsically unstructured, providing the flexibility necessary for intra- and/or inter-molecular N–C interactions. However, this flexibility also makes it difficult to conduct X-ray crystallography on CTTNBP2. We have repeatedly tried to purify full-length recombinant CTTNBP2 protein. Perhaps due to its unstructured nature, the recombinant protein is unstable in bacterial expression systems. Consequently, as yet, it is completely unknown how the N- and C-terminal regions of CTTNBP2 interact with each other and how the N-terminal region of CTTNBP2 can regulate the interaction between the C-terminal P-rich domain and cortactin.

Here, we have reported that a N–C terminal interaction facilitates the interaction of CTTNBP2 C-terminal with cortactin, with this latter being disrupted by M120I mutation. This kind of facilitation is not rare in cytoskeleton regulators. For instance, N-WASP is a core signal transducer that receives signal from CDC42 and directs Arp2/3-dependent actin polymerization [1]. The intramolecular N–C terminal interaction of N-WASP occludes the Arp2/3 binding site to inhibit actin polymerization [16]. Similarly, the microtubule binding and stabilizing property of mPar3 is facilitated by an intramolecular N–C terminal interaction [3]. Intermolecular oligomerization of mPar competes for the intramolecular N–C terminal interaction, exposing the microtubule-binding domain of mPar3 [3]. Our study on CTTNBP2 provides a new example of a cytoskeleton regulator that is modulated by its N–C interaction.

Although the D570Y mutation also reduced dendritic spine density, the underlying mechanism is different from those of the M120I and R533* mutations. Its higher affinity for microtubule results in this variant preferentially staying in the dendritic shaft rather than moving into dendritic spines. The reduction of CTTNBP2 protein levels at dendritic spines resembles the outcomes of *Cttnbp2* knockdown or knockout, resulting in lower dendritic spine density. However, apart from dendritic spine deficits, the binding of D570Y mutant protein to microtubule along the dendritic shaft and consequently increased microtubule stability may contribute to other phenotypes arising from D570Y mutation. This latter possibility requires further investigation. Interestingly, residue D570 is located very close to the C-terminal end of the short form of CTTNBP2 (630 amino acid residues) [5]. Our previous data showed that the middle unstructured region of CTTNBP2 is required for association with microtubule [21]. Thus, the C-terminal region of CTTNBP2 may also regulate the protein interactions mediated by its middle region, which likely relates to the intrinsically unstructured nature of CTTNBP2. Our study implies that the protein structure of CTTNBP2 is very flexible and exhibits complex self-interactions and regulation.

In this report, we have primarily focused on dendritic spine defects, with the R533* and M120I mutations resulting in the most severe phenotypes. For other investigated mutations, such as R42W and G342R, their impact on dendritic spine density was much milder. Unlike for WT protein that slightly increased dendritic spine density, overexpression of R42W and G342R mutants was unable to do so. This outcome indicates that R42W and G342R likely represent loss-of-function mutations. Apart from its influence on dendritic spines, CTTNBP2 also regulates dendritic arborization [21]. Since CTNNBP2 is also present along the axonal shaft, it would also be meaningful to investigate if these ASD-linked mutations influence dendritic arborization and/or axonal growth, which may also contribute to ASD phenotypes.

Taken together with our previous study [20], we have now analyzed the behavioral features of *Cttnbp2*^−*/*−^, *Cttnbp2*^+*/*−^, R533* and M120I mutant mice. Reduced social interactions with strangers in reciprocal social interaction and three-chamber tests are common defects shared among all of these examined mutant mouse lines. Since defective social interaction is one of the core symptoms of ASD, our mouse studies support that CTTNBP2 mutation is at least partially responsible for ASD. Despite these common social defects, other behavioral deficits are not exhibited by all of the examined mouse lines. For instance, *Cttnbp2*^−*/*−^ mice exhibit the most severe behavioral abnormalities, including hyperactive locomotion, anxiolytic behaviors in elevated plus maze and marble burying tests, and impaired contextual memory in novel object recognition [20]. However, *Cttnbp2*^+*/*−^, R533* and M120I mutant mice did not display any noticeable phenotype in terms of locomotion or anxiety. Thus, it would be informative to correlate patients’ mutations and symptoms with the phenotypes displayed by our mouse models. Such analysis would provide useful information as to whether mouse behavioral assays, at least for *Cttnbp2* mutant mice, are relevant to patients’ conditions and could serve as appropriate models for etiological and therapeutic studies.

## Supplementary information

**Additional file 1: Fig. S1** Amino acid sequence alignment of human, rat and mouse CTTNBP2.

**Additional file 2: Table S1** contains all statistical methods and results, except Fig. 5B.

**Additional file 3: Table S2** contains the statistical results of Fig. 5B.

## Data Availability

Data generated or analyzed during this study are included in this published article and its supplementary information files.
