# Supplementary material for: Autism-linked mutations of CTTNBP2 reduce social interaction and impair dendritic spine formation via diverse mechanisms
Source: Acta Neuropathol Commun. 2020 Nov 9;8:185. doi: 10.1186/s40478-020-01053-x (PMC7654188; doi:10.1186/s40478-020-01053-x)
Supplement: Supplementary file 1 — Additional file 1: Fig. S1 Amino acid sequence alignment of human, rat and mouse CTTNBP2. [file 40478_2020_1053_MOESM1_ESM.pdf]

Supplemental Figure and Figure Legend (By Shih et al.)

```
Human          1    MATDGASCEPDLSRAPEDAAGAAAEAAKKEFDVDTLSKSELRMLLSVMEGELEARDLVIEALRARRKEVFIQERYGRFNL    80
Rat (84%)      1    MATDSASCEPDLSRAPGDAEGATAEAAKKEFDVDTLSKSELRMLLSVMEGELEARDLVIEALRARRKEVFIQERYGRFNL    80
Mouse (84%)    1    MATDSASCEPDLSRTPGDTEGATAEAAKKEFDVDTLSKSELRMLLSVMEGELEARDLVIEALRARRKEVFIQERYGRFNL    80

Human          81   NDPFLALQRDYEAGAGDKEKkPVCTNPLSILEAVMAHCKKMQERMSAQLAAAESRQKKLEMEKLQLQALEQEHKKLAARL    160
Rat (84%)      81   NDPFLALQRDYEAGAGDKEK-PVCTNPLSILEAVMAHCRKMQERMSAQLAAAESRQKKLEMEKLQLQALEQEHKKLAAHL    159
Mouse (84%)    81   NDPFLALQRDYEAGPGDKEK-PVCTNPLSILEAVMAHCRKMQERMSAQLVAAESRQKKLEMEKLQLQALEQEHKKLAAHL    159

Human          161  EEERGKNKQVVLMLVKECKQLSGKVIEEAQKLEDVMAKLEEEKKKTNELEEELSAEKRRSTEMEAQMEKQLSEFDTEREQ    240
Rat (84%)      160  EEERGKNKHVVLMLVKECKQLSGKVVEEAQKLEEVMVKLEEEKKKTSELEDQLSAEKQRSAGMEAQLEKQLFEFDTEREQ    239
Mouse (84%)    160  EEERGKNKHVVLMLVKECKQLSGKVVEEAQKLEEVMAQLEEEKKKTSELEEQLSAEKQRSSGMEAQLEKQLSEFDTEREQ    239

Human          241  LRAKLNREEAHTTDLKEEIDKMRKMIEQLKRGSDSKPSLSLPRKTKDRRLVSISVGTEGTVTRSVACQTDLVTENADHMK    320
Rat (84%)      240  LRAKLTREEAHTTDLKEEIDKMKKMMEQMKKGNDGKPGLSLPRKTKDKRLASISVATEGPVTRSVACQTDVVTESTDPVK    319
Mouse (84%)    240  LRAKLSREEAHTTDLKEEIDKMKKMMEQMKKGSDGKPGLSLPRKTKDKRLASISVATEGPVTRSVACQTDVVTESTDPVK    319

Human          321  KLPLTMPVKPSTGSPLVSANAKGSVCTSATMARPGIDRQASYGDLiGASVPAFPPPSANKIEENGPSTGSTPDPTSSTPP    400
Rat (84%)      320  KLPLSVPIKPSTGSPLVSTNTKGNVGPSALLIRPGIDRQASHSDL-GPSPPTALPSSASRIEENGPSAGNAPDLSNSTPS    398
Mouse (84%)    320  KLPLTVPIKPSTGSPLVPTNTKGNVGPSALLIRPGIDRQSSHSDL-GPSPPTALPSSANRIEENGPSTGNAPDLSNSTPS    398

Human          401  LPSNAAPPTAQTPGIAPQN-SQAPPMHSLHSPCANTslHPGLNPRIQAARFRFQGNANDPDQNGNTTQSPPSRDVSPTSR    479
Rat (84%)      399  TPSGTAPAAAQTLGAAPQNHSQAPPVHSLHSPCANT--HPGLNPRIQAARFRFQGNANDPDQNGNNTQSPPSRDVSPTSR    476
Mouse (84%)    399  TPSSTAPAAAQTPGTAPQNHSQAPTVHSLHSPCANT--HPGLNPRIQAARFRFQGNANDPDQNGNNTQSPPSRDVSPTSR    476

Human          480  DNLVAKQLARNTVTQALSRFTSPQAGAPSRPGVPPTGDVGTHPPVGRTSLKTHGVARVDRGNPPPIPPKKPGLSQTPSPP    559
Rat (84%)      477  DNLVAKQLARNTVTQALSRFTSPQAGASSRLGASPGGDAGTCPPVGRTGLKTPGAARVDRGNPPPIPPKKPGLSQTPSPP    556
Mouse (84%)    477  DNLVAKQLARNTVTQALSRFTSPQAGASSRLGVSPGGDAGTCPPVGRTGLKTPGAARVDRGNPPPIPPKKPGLSQTPSPP    556

Human          560  HPQL[7]RASNTGAKVDNKTVASTPSSLPQGNRVINEENLPKSSSPQLPPKPSIDLTVAPAGCAVSALATSQVGAWPAAT    640
Rat (84%)      557  HPQL   RASNAGAKVDNKIVASPPSTLPQGTKVVNEENVPKSSSPQLPPKPSIDLTVASAGCPVSALATSQVGAWPAET    630
Mouse (84%)    557  HPQL   RASNAGAKVDNKIVASPPSTLPQGTKVVNEENVPKSSSPQLPPKPSIDLTVAPAGCPVSALATSQAGHPP---    630
```

Supplemental Figure S1. Amino acid sequence alignment of the short form of CTTNBP2 from human, rat and mouse. Boxes indicate the ASD-linked mutations studied in this report. Sequence identities of human vs. mouse and human vs. rat are both 84%. The alignment was performed using NCBI BLAST multiple sequence alignment.
