# Supplementary material for: Autism-linked mutations of CTTNBP2 reduce social interaction and impair dendritic spine formation via diverse mechanisms
Source: Acta Neuropathol Commun. 2020 Nov 9;8:185. doi: 10.1186/s40478-020-01053-x (PMC7654188; doi:10.1186/s40478-020-01053-x)
Supplement: Supplementary file 2 — Additional file 2: Table S1 contains all statistical methods and results, except Fig. 5B. [file 40478_2020_1053_MOESM2_ESM.pdf]

Figure	Experiment	Samples	N	n	Mean	Std. Deviation	Std. Error of Mean	Test			p-value	Significance?
1c	Spine number in 20 µm	Vector	25	146	9.137	3.926	0.3249	Kruskal-Wallis test	Dunn's multiple comparisons test			
	N, neuron number; n, dendrite number	WT	30	216	10.16	3.782	0.2573			WT vs. Vector	0.0491	*
		R42W	23	165	9.036	4.475	0.3484			WT vs. R42W	0.0223	*
		A112T	34	205	10.43	4.259	0.2975			WT vs. A112T	>0.9999	ns
		M120I	33	178	7.455	3.901	0.2924			WT vs. M120I	<0.0001	***
		G342R	33	204	8.887	4.398	0.3079			WT vs. G342R	0.0055	**
		P353A	28	183	11	3.546	0.2621			WT vs. P353A	0.2658	ns
		R533*	42	258	7.678	4.383	0.2729			WT vs. R533*	<0.0001	***
		D570Y	37	233	10.09	4.422	0.2897			WT vs. D570Y	>0.9999	ns
										Vector vs. WT	0.0491	#
										Vector vs. R42W	>0.9999	ns
										Vector vs. A112T	0.0239	#
										Vector vs. M120I	0.0101	#
										Vector vs. G342R	>0.9999	ns
										Vector vs. P353A	<0.0001	###
										Vector vs. R533*	0.0171	#
										Vector vs. D570Y	0.1986	ns
1d	mEPSC frequency	WT	21		2.604	1.584	0.3457	Ordinary one way ANOVA test F(2, 51) = 8.390 p=0.0007	Bonferroni's multiple comparisons test	WT vs. M120I	0.0205	*
	N, neuron number	M120I	15		1.541	0.8394	0.2167			WT vs. R533*	0.0005	***
		R533*	18		1.102	0.8032	0.1893					
	mEPSC amplitude	WT	21		14.02	4.448	0.9706	Ordinary one way ANOVA test F (2, 51) = 3.064 p=0.0554	Bonferroni's multiple comparisons test	WT vs. M120I	0.0545	ns
		M120I	15		11.25	2.936	0.7582			WT vs. R533*	0.1324	ns
		R533*	18		11.84	2.971	0.7004					
3a	Cortactin rescues MI and R* expressing neuron	Vector/Vector	31	93	7.495	3.332	0.3455	Two way ANOVA		Vector vs. CTTN	<0.0001	***
	Spine number in 20 µm	CTTN/Vector	25	75	9.84	4.159	0.4803	F (1, 613) = 97.75, P<0.0001				
	N, neuron number; n, dendrite number	Vector/WT	27	81	11.11	4.047	0.4496					
		CTTN/WT	25	75	13.76	4.846	0.5595					
		Vector/M120I	26	78	6.821	4.015	0.4547					
		CTTN/M120I	18	54	11.24	3.909	0.532					
		Vector/R533*	28	84	6.798	3.715	0.4053					
		CTTN/R533*	27	81	10.27	4.096	0.4551					
3b	Relative CTTN signal in dendritic spine	GFP	30	264	1.86	0.7348	0.0452	Two-tailed Mann Whitney test		GFP vs. GFP-Prich	<0.0001	***
	N, dendrite number; n, spine number	GFP-Prich	45	260	1.571	0.7379	0.0458					
3c	Spine number in 20 µm	GFP	28	96	11.16	3.873	0.3952	Two tailed unpaired t test	t=7.514, df=184	GFP vs. GFP-Prich	<0.0001	***
	N, neuron number; n, dendrite number	GFP-Prich	30	90	7.056	3.549	0.3741					
3d	mEPSC frequency	GFP	8		2.665	1.343	0.4748	Two tailed unpaired t test	t=3.081, df=13	GFP vs. GFP-Prich	0.0088	**
	N, neuron number	GFP-Prich	7		0.9871	0.5424	0.205					
	mEPSC amplitude	GFP	8		15.39	5.464	1.932	Two tailed unpaired t test	t=1.740, df=13	GFP vs. GFP-Prich	0.1054	ns
	N, neuron number	GFP-Prich	7		11.64	1.674	0.6327					
5d	Relative CTTNBP2 intensity	WT 20 ug	4		1	0.188	0.094	Two way ANOVA	Bonferroni's multiple comparisons test	WT vs. D570Y 20 ug	<0.0001	***
	N, independent experiments	WT 10 ug	4		0.4764	0.2525	0.1263	Two way ANOVA	Bonferroni's multiple comparisons test	WT vs. D570Y 10 ug	0.0007	***
		WT 5 ug	4		0.1673	0.0536	0.0268	Two way ANOVA	Bonferroni's multiple comparisons test	WT vs. D570Y 5 ug	0.0278	*
		D570Y 20 ug	4		2.3235	0.344	0.172					
		D570Y 10 ug	4		1.5692	0.5015	0.2507					
		D570Y 5 ug	4		0.8603	0.4546	0.2273					
5e	Acetyl-tubulin/tubulin ratio	WT Nocodazole	15		0.7666	0.2405	0.0621	Two-tailed Mann Whitney test		WT vs. D570Y	0.0128	*
	N, neuron number	D570Y Nocodazole	15		1.109	0.3746	0.0967					
6b	Spine number in 20 µm	Ctrl-miR	15		12.27	3.035	0.7836	Two way ANOVA	Bonferroni's multiple comparisons test	Ctrl-miR: WT vs. DY	0.0305	*
	N, neuron number	BP2-miR	15		8.2	1.474	0.3805			BP2-miR: WT vs. DY	<0.0001	***
		WT/Ctrl-miR	40		12.08	2.177	0.3442					
		D570Y/Ctrl-miR	38		10.89	2.357	0.3824					
		WT/BP2-miR	38		10.63	1.992	0.3232					
		D570Y/BP2-miR	30		8.067	1.874	0.3422					
6c-d	Myc-tag signal 0-0.5 µm	WT/Ctrl-miR	35		338.2	215.7	36.46	Two way ANOVA	Bonferroni's multiple comparisons test	Ctrl + WT vs. Ctrl + D570Y	***	0.0001
	N, neuron number	D570Y/Ctrl-miR	35		258.4	146.6	24.78	F (3, 816) = 20.26, P<0.0001		BP2-miR + WT vs. BP2-miR + D570Y	***	<0.0001
		WT/BP2-miR	35		334.3	205.2	34.68	Two way ANOVA	Bonferroni's multiple comparisons test	Ctrl + WT vs. Ctrl + D570Y	***	0.0002
		D570Y/BP2-miR	35		218	131.7	22.27	F (3, 800) = 15.27, P<0.0001		BP2-miR + WT vs. BP2-miR + D570Y	***	<0.0001
	Myc-tag signal 1-1.5 µm	WT/Ctrl-miR	35		173.8	167.7	28.35	Two-tailed Mann Whitney test		Summed Ctrl-miR WT vs. DY/0-0.5 µm	0.1159	ns
	N, neuron number	D570Y/Ctrl-miR	35		266.8	206.3	34.86	Two-tailed Mann Whitney test		Summed BP2-miR WT vs. DY/0-0.5 µm	0.0154	*
		WT/BP2-miR	35		186	136.6	23.08	Two-tailed Mann Whitney test		Summed Ctrl-miR WT vs. DY/1-1.5 µm	0.0088	**
		D570Y/BP2-miR	35		285.8	268.5	45.38	Two-tailed Mann Whitney test		Summed BP2-miR WT vs. DY/1-1.5 µm	0.1704	ns
7f	Spine number in 10 µm	+/+	4	40	11.55	2.882	0.4557	Two-tailed Mann Whitney test		+/+ vs M120I	0.0004	***
		M120I	4	40	9.267	2.771	0.4381					
	Spine width	+/+	4	40	0.4558	0.0406	0.0064	Two-tailed unpaired t test	t=5.848, df=78	+/+ vs M120I	<0.0001	***
		M120I	4	40	0.3992	0.0458	0.0072					
	Spine length	+/+	4	40	0.7523	0.0877	0.0139	Two-tailed unpaired t test	t=3.907, df=78	+/+ vs M120I	0.0002	***
	N, animal number; n, neuron number	M120I	4	40	0.6796	0.0785	0.0124					
7i	Spine number in 10 µm	+/+	3	30	9.733	2.212	0.4038	Two-tailed unpaired t test	t=3.018, df=58	+/+ vs D570Y	0.0038	**
		D570Y	3	30	7.8	2.724	0.4974					
	Spine width	+/+	3	30	0.443	0.0515	0.0094	Two-tailed Mann Whitney test		+/+ vs D570Y	0.4404	ns
		D570Y	3	30	0.4518	0.0515	0.0094					
	Spine length	+/+	3	30	0.7043	0.0906	0.0165	Two-tailed Mann Whitney test		+/+ vs D570Y	0.1872	ns
	N, animal number; n, neuron number	D570Y	3	30	0.7215	0.1006	0.0184					
8b	Open field total distance (cm)	+/+	10		4253	1185	374.7	Two-tailed unpaired t test	t=1.571, df=18	+/+ vs M120I	0.1336	ns
		M120I	10		4261	809.6	256					
	Open field Tcent/Tcorn ratio	+/+	10		0.2202	0.1007	0.0319	Two-tailed unpaired t test	t=0.01899, df=18	+/+ vs M120I	0.9851	ns
	N, animal number	M120I	10		0.3093	0.1485	0.047					
8c	Elevated plus maze percent of time	+/+ OP	10		13.41	6.217	1.966	Two-tailed unpaired t test	t=0.2188, df=18	+/+ OP vs M120I OP	0.8293	ns
		M120I OP	10		71.02	9.154	2.895					
		+/+ CL	10		12.78	6.574	2.079	Two-tailed unpaired t test	t=0.1049, df=18	+/+ CL vs M120I CL	0.9177	ns
	N, animal number	M120I CL	10		71.52	12.25	3.873					
8d	Reciprocal social interaction time (RSI)	+/+	10		85.7	60.14	19.02	Two-tailed Mann Whitney test		+/+ vs M120I	0.0139	*
	N, animal number	M120I	10		38.5	34.02	10.76					
8e	Three chamber test sociability	+/+ Ob	10		43.6	19.02	6.013	Two-tailed paired t test	t=6.298, df=9	+/+ Ob vs +/+ S1	0.0001	***
	Interaction time	+/+ S1	10		129.7	38.97	12.32					
	N, animal number	M120I Ob	10		65.7	18.09	5.719	Two-tailed paired t test	t=5.325, df=9	M120I Ob vs M120I S1	0.0005	***
		M120I S1	10		104.7	24.99	7.902					
	Three chamber test sociability	+/+	10		86.1	43.23	13.67	Two-tailed unpaired t test	t=3.131, df=18	+/+ vs M120I	0.0058	**
	S1-O	M120I	10		38	22.16	7.006					
	Three chamber test sociability	+/+	10		0.4912	0.1778	0.0562	Two-tailed unpaired t test	t=3.752, df=18	+/+ vs M120I	0.0015	**
	Preference index	M120I	10		0.2295	0.1305	0.0413					
8f	Three chamber test novety preference	+/+ S1	10		49.8	18.64	5.893	Two-tailed paired t test	t=4.364, df=9	+/+ S2 vs +/+ S1	0.0018	**
	Interaction time	+/+ S2	10		106.5	28.37	8.972					
	N, animal number	M120I S1	10		65.4	25.1	7.938	Two-tailed paired t test	t=0.7810, df=9	M120I S2 vs M120I S1	0.4549	ns
		M120I S2	10		77.1	27.28	8.627					
	Three chamber test novety preference	+/+	10		56.7	41.09	12.99	Two-tailed unpaired t test	t=2.345, df=18	+/+ vs M120I	0.0307	*
	S2-S1	M120I	10		10.7	46.48	14.7					
	Three chamber test novety preference	+/+	10		0.3562	0.2384	0.0754	Two-tailed Mann Whitney test		+/+ vs M120I	0.0355	*
	Preference index	M120I	10		0.0824	0.3116	0.0986					
8g	dCA1 CFOS+ cell number/mm2	+/+	6		119.9	44.3	18.08	Two-tailed Mann Whitney test		+/+ vs M120I	0.026	*
		M120I	6		73.76	22.6	9.226					
	dCA2 CFOS+ cell number/mm2	+/+	6		63.17	22.91	9.352	Two-tailed unpaired t test	t=2.732, df=10	+/+ vs M120I	0.0211	*
		M120I	6		29.01	20.33	8.298					
	dCA3 CFOS+ cell number/mm2	+/+	6		154.4	37.2	15.19	Two-tailed unpaired t test	t=2.240, df=10	+/+ vs M120I	0.049	*
		M120I	6		116.8	17.34	7.08					
	DG CFOS+ cell number/mm2	+/+	6		143.4	30.35	12.39	Two-tailed unpaired t test	t=1.720, df=10	+/+ vs M120I	0.1162	ns
		M120I	6		115.2	26.14	10.67					
	RSP CFOS+ cell number/mm2	+/+	6		207.7	111.7	45.59	Two-tailed unpaired t test	t=1.513, df=10	+/+ vs M120I	0.1613	ns
		M120I	6		130.7	55.45	22.64					
	MO CFOS+ cell number/mm2	+/+	6		257.3	73.08	29.83	Two-tailed unpaired t test	t=1.403, df=10	+/+ vs M120I	0.1908	ns
		M120I	6		191.8	87.85	35.87					

SOp CFOS+ cell number/mm2	+/+	6	303.6	77.18	31.51	Two-tailed unpaired t test	t=1.244, df=10	+/+ vs M120I	0.2419	ns
	M120I	6	228.2	126.8	51.78					
BLA CFOS+ cell number/mm2	+/+	6	81.14	33.49	13.67	Two-tailed unpaired t test	t=3.308, df=10	+/+ vs M120I	0.0079	**
N, animal number	M120I	6	32.12	14.01	5.721					
