# Supplementary material for: Autism-linked mutations of CTTNBP2 reduce social interaction and impair dendritic spine formation via diverse mechanisms
Source: Acta Neuropathol Commun. 2020 Nov 9;8:185. doi: 10.1186/s40478-020-01053-x (PMC7654188; doi:10.1186/s40478-020-01053-x)
Supplement: Supplementary file 3 — Additional file 3: Table S2 contains the statistical results of Fig. 5B. [file 40478_2020_1053_MOESM3_ESM.pdf]

µm	WT (n=56)		DY (n=44)			
	Mean	SD	Mean	SD	P value	Summary
0	0.9265	0.1667	1.237	0.2278	P > 0.05	ns
1	0.9106	0.1618	1.3469	0.1961	P<0.01	**
2	1.0236	0.0996	1.5564	0.1369	P<0.001	***
3	1.0264	0.0356	1.6213	0.0896	P<0.001	***
4	0.9724	0.0247	1.6209	0.1531	P<0.001	***
5	0.9372	0.0494	1.6046	0.1532	P<0.001	***
6	0.9975	0.0125	1.6514	0.1726	P<0.001	***
7	1.0245	0.041	1.739	0.149	P<0.001	***
8	1.0878	0.0759	1.7252	0.1245	P<0.001	***
9	1.0238	0.0081	1.6767	0.0804	P<0.001	***
10	1.0289	0.0567	1.667	0.0009	P<0.001	***
11	1.0547	0.1046	1.5524	0.0219	P<0.001	***
12	1.0501	0.0033	1.5142	0.0832	P<0.001	***
13	1.0366	0.0958	1.4257	0.0978	P<0.01	**
14	1.0395	0.1406	1.3658	0.0594	P > 0.05	ns
15	1.0168	0.0263	1.2608	0.0465	P > 0.05	ns
16	1.0945	0.0403	1.1914	0.047	P > 0.05	ns
17	1.1104	0.1261	1.1412	0.0722	P > 0.05	ns
18	1.1262	0.0541	1.0823	0.0181	P > 0.05	ns
19	1.0632	0.0023	1.0175	0.0157	P > 0.05	ns
20	1.0013	0.0655	0.9705	0.0495	P > 0.05	ns
21	0.8627	0.1923	0.904	0.0866	P > 0.05	ns
22	1.0187	0.2001	0.8225	0.1595	P > 0.05	ns
23	0.9162	0.1842	0.7066	0.1743	P > 0.05	ns
24	0.8552	0.1494	0.6977	0.049	P > 0.05	ns
25	1.1209	0.0589	0.677	0.078	P<0.001	***
26	1.066	0.1502	0.6291	0.0668	P<0.01	**
27	0.8344	0.1413	0.5453	0.0235	P > 0.05	ns
28	0.9192	0.0568	0.543	0.0632	P < 0.05	*
29	0.9032	0.0329	0.5308	0.0154	P < 0.05	*
30	0.998	0.1092	0.5207	0.0162	P<0.001	***
31	1.1496	0.0578	0.5359	0.026	P<0.001	***
32	0.9379	0.0457	0.5362	0.0323	P<0.01	**
33	0.903	0.0811	0.493	0.0115	P<0.01	**
34	0.8278	0.118	0.5065	0.02	P > 0.05	ns
35	0.9624	0.1132	0.444	0.0131	P<0.001	***
36	1.0301	0.0083	0.4813	0.0401	P<0.001	***
37	1.0565	0.0973	0.5059	0.0457	P<0.001	***
38	0.9222	0.0096	0.4932	0.0595	P<0.01	**
39	0.9229	0.0461	0.4935	0.076	P<0.01	**
40	1.021	0.1155	0.459	0.1679	P<0.001	***
